# Machine Learning-Driven Identification of Key Environmental Factors Influencing Fiber Yield and Quality Traits in Upland Cotton

**DOI:** 10.3390/plants14132053

**Published:** 2025-07-04

**Authors:** Mohamadou Souaibou, Haoliang Yan, Panhong Dai, Jingtao Pan, Yang Li, Yuzhen Shi, Wankui Gong, Haihong Shang, Juwu Gong, Youlu Yuan

**Affiliations:** 1 State Key Laboratory of Cotton Bio-Breeding and Integrated Utilization, Institute of Cotton Research, Chinese Academy of Agricultural Sciences, Anyang 455000, China; raoubil01@gmail.com (M.S.); yanhaoliang@caas.cn (H.Y.); panjingtao@caas.cn (J.P.); liyang06@caas.cn (Y.L.); shiyuzhen@caas.cn (Y.S.); gongwankui@caas.cn (W.G.); shanghaihong@caas.cn (H.S.); 2 Zhengzhou Research Base, State Key Laboratory of Cotton Bio-Breeding and Integrated Utilization, School of Agricultural Sciences, Zhengzhou University, Zhengzhou 450001, China; 3 College of Computer Science and Information Engineering, Anyang Institute of Technology, Anyang 455000, China; daipanhong@126.com; 4 Kashi Academy of Advanced Agricultural Sciences, Kashi 844000, China

**Keywords:** cotton, machine learning, genotype environment interaction, SHAP interpretation, environmental factors

## Abstract

Understanding the influence of environmental factors on cotton performance is crucial for enhancing yield and fiber quality in the context of climate change. This study investigates genotype-by-environment (G×E) interactions in cotton, using data from 250 recombinant inbred lines (CCRI70 RILs) cultivated across 14 diverse environments in China’s major cotton cultivation areas. Our findings reveal that environmental effects predominantly influenced yield-related traits (boll weight, lint percentage, and the seed index), contributing to 34.7% to 55.7% of their variance. In contrast fiber quality traits showed lower environmental sensitivity (12.3–27.0%), with notable phenotypic plasticity observed in the boll weight, lint percentage, and fiber micronaire. Employing six machine learning models, Random Forest demonstrated superior predictive ability (R^2^ = 0.40–0.72; predictive Pearson correlation = 0.63–0.86). Through SHAP-based interpretation and sliding-window regression, we identified key environmental drivers primarily active during mid-to-late growth stages. This approach effectively reduced the number of influential input variables to just 0.1–2.4% of the original dataset, spanning 2–9 critical time windows per trait. Incorporating these identified drivers significantly improved cross-environment predictions, enhancing Random Forest accuracy by 0.02–0.15. These results underscore the strong potential of machine learning to uncover critical temporal environmental factors underlying G×E interactions and to substantially improve predictive modeling in cotton breeding programs, ultimately contributing to more resilient and productive cotton cultivation.

## 1. Introduction

Cotton is a major cash crop and a vital raw material for the textile industry, significantly contributing to the economies of many countries. Driven by global population growth and increasing consumer demand, the gap between supply and demand for high-quality cotton fiber continues to widen [1]. Improving the production of quality cotton, therefore, represents a significant challenge for modern cotton agriculture. Although genetic improvement has yielded considerable progress, environmental factors and genotype–environment (G×E) interactions remain crucial determinants of fiber yield and quality [2].

To understand this complexity, it is important to precisely define the concept of G×E: it corresponds to the differential response of a genotype for a given trait according to environmental conditions [3]. According to [4], this interaction, a fundamental element of plant breeding programs, involves the simultaneous optimization of three complementary parameters: absolute yield potential (comparison with controls), specific adaptation (environments favoring optimal expression), and phenotypic stability (regularity of performance across various environments). Cotton productivity thus results from the dynamic interaction between the genetic potential of cultivars and environmental conditions. While certain agronomic parameters (fertilization, stand density, and pest control) can be controlled, climatic factors (solar radiation, wind, rainfall, temperature, and relative humidity) present intrinsic spatial and temporal variability, and remain non-adjustable in the short term [5].

Studies conducted in different regions have shown that location and environment are responsible for up to 90% of the variation in cotton yield [6]. Climatic factors such as temperature, rainfall, solar radiation, and humidity significantly affect the production of cotton fiber yield and quality. Sawan [7] reported a negative correlation between reproductive development and both evaporation and sunlight duration. The underlying physiological mechanisms involve the inhibition of diurnal photosynthetic activity and increased nocturnal respiration, leading to a decrease in available carbon assimilates without diurnal photosynthetic compensation [8,9]. These metabolic disturbances result in the increased abortion of reproductive organs, a decreased number of seeds per capsule, and altered fiber development. Under low radiation, ethylene accumulation accelerates boll maturation, causing premature fruit drop and yield decline [10].

Temperature plays a pivotal role in reproductive development by regulating pollen germination, pollen tube growth, and fruiting processes [11,12,13]. For optimal development, cotton requires specific thermal conditions: at least 15 °C for germination, between 21 °C and 27 °C for vegetative growth, and between 27 °C and 32 °C during fruiting [14]. Above 40 °C, cellular metabolism slows down, affecting growth and yield, while below 5 °C, tissues sustain damage, reducing fiber quality [14]. While yield components generally improve with lower average temperatures, fiber length, uniformity, strength, and micronaire increase with higher temperatures, especially during the daytime [15].

Cotton production remains limited by both water availability and temperature. In Xinjiang, China, Li et al. [16] demonstrated that rainfall is a major factor influencing cotton stalk weight and fiber percentage, explaining up to 85.7% of changes in lint percentage. Water deficit limits boll formation and disrupts metabolic processes such as photosynthesis and ATP synthesis, leading to biomass reduction [17].

Climate change has increased the unpredictability of yield and fiber quality in cotton production. Over the past fifty years, rising temperatures during the cotton-growing season have been observed across all major producing regions [18]. Extreme weather events—heat waves, droughts, and intense rainfall—are becoming more frequent, though with regional variation, further intensifying climate heterogeneity [19]. These disruptions directly impact cotton growth and development, elevating systemic production risks.

To address this challenge, in-depth analyses of genotype–environment (G×E) interactions are essential for identifying genotypes best adapted to specific environmental contexts. A powerful tool in this regard is the use of recombinant inbred lines (RILs). Derived from the repeated selfing of F1 hybrids, RILs are genetically stable and reproducible, allowing robust phenotyping and precise QTL and G×E analysis. To apply this strategy, researchers developed a RIL population from the high-yield, high-fiber quality variety CCRI70, an elite cultivar approved in China in 2008. This population, comprising 250 lines, was specifically created to uncover alleles associated with superior fiber traits and to support the genetic improvement of upland cotton [20,21].

Linear regression represents a commonly used method to analyze relationships between independent variables (environmental factors) and a dependent variable (the phenotypic trait) in the context of phenotypic plasticity [22]. This approach quantifies the effect of environmental variations on trait adaptability and is particularly valuable in agricultural studies due to its simplicity in application and interpretation. Linear regression helps identify the most influential environmental factors affecting the phenotypic plasticity of specific traits. Complementary to this, advanced statistical models allow for more robust analysis of genotype–environment (G×E) interactions. For instance, mixed models (ANOVA with fixed and random effects) evaluate both genetic and environmental variation as well as their interactions, providing insights into how differently genotypes respond to environmental variations [23].

Additionally, biadditive or bilinear models, such as the AMMI (additive main effects and multiplicative interaction) model and the GGE (genotype + genotype × environment interaction) model, are employed to model and analyze complex interactions between genotypes and environments, especially in relation to phenotypic plasticity [24]. However, due to the complex non-linear relationship between genotype and environment interactions, traditional analysis methods often miss substantial amounts of important information.

Recent advances in machine learning (ML) offer powerful alternatives for modeling complex G×E interactions. It can automatically learn data patterns, optimize model parameters, and is more inclusive of non-linear relationships [25]. Random Forests are a machine learning technique renowned for their ability to evaluate variable importance in classification and regression models [26]. They are widely used to analyze genotype–environment (G×E) interactions across various crops, including wheat, maize, and cotton. These algorithms allow for modeling complex relationships between genetic and environmental variables, thereby facilitating the prediction of phenotypic performance.

For wheat, researchers have employed Random Forests to assess G×E interactions, predicting yield across diverse environments and identifying the most performant genotypes [27]. Similarly, in maize, studies have integrated multi-omic approaches with Random Forests to better understand G×E interactions. These analyses have enabled the prediction of maize hybrid performance across various environments, incorporating genomic, transcriptomic, and proteomic data [28]. As for cotton, Random Forests have been utilized to predict yield and fiber quality based on environmental variables such as temperature, perception, and nutrient availability [29]. These models have facilitated the identification of cotton genotypes best suited to specific conditions, thus optimizing varietal selection.

While numerous studies have examined the impact of environmental conditions on cotton yield and fiber quality, the majority have primarily focused on a limited number of climatic factors [18,30,31]. Few studies have successfully deciphered the effects of various climatic conditions on phenotypic traits at different growth stages of the plant across diverse environments. Therefore, a comprehensive approach that considers the influence of meteorological factors, including temperature, perception, humidity, radiation, and wind speed over multiple years and locations based on ML, is essential for a thorough understanding of environmental effects on cotton yield and quality parameters. This study aims to address three main objectives: (1) to evaluate significant variations in cotton fiber quality across years in response to regional climatic conditions; (2) to identify key environmental factors responsible for these variations and their critical temporal windows; and (3) to optimize prediction models by integrating these key environmental factors to better understand how genotypes respond to environmental variations.

## 2. Materials and Methods

### 2.1. Materials

This study focused on recombinant inbred lines (RILs) derived from the transgenic cotton variety CCRI70. This variety was developed by crossing two parental lines: sGK156, known for its resistance to insects and fungal diseases, and 901-001, recognized for its exceptional fiber quality. CCRI70 is notable for its high yield, disease resistance, and superior fiber quality (length: 32.5 mm; strength: 33.5 cN/tex; micronaire: 4.3). Further details are available in [32]. The development of these RILs commenced in 2011 with initial crosses, followed by multiple cycles of self-fertilization across generations (F2 to F7), conducted in the Henan and Hainan provinces in China. This meticulous process facilitated the selection of stable lines, which were subsequently used for genomic genotyping and to evaluate phenotypic traits related to fiber yield and quality [20]. Genotyping and the analysis of single-nucleotide polymorphism (SNP) markers of the population can be found in Jiang’s research [32]

### 2.2. Experimental Design

**Figure 1:**
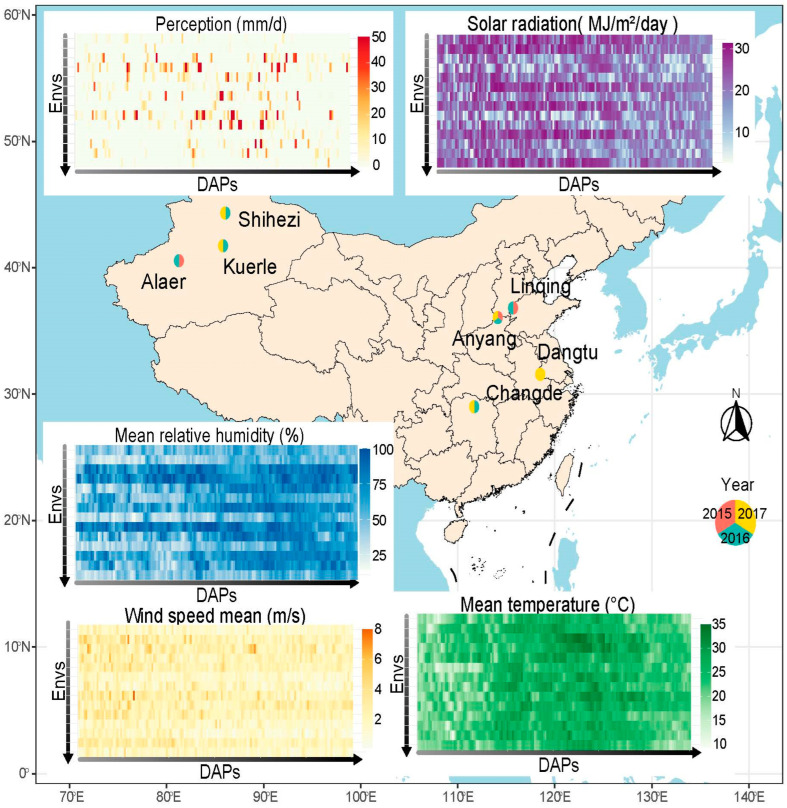
Perception (pcp), radiation (radn), mean temperature (tmean), mean relative humidity (rhmean), and mean wind speed (wsmean) conditions during the 150-day period after cotton sowing across the 14 experimental environments. The order of the 14 environments from top to bottom is 17SHZ, 17KEL, 17DT, 17CD, 17AY, 16SHZ, 16LQ, 16KEL, 16CD, 16AY, 16ALE, 15LQ, 15AY, and 15ALE.

Between 2015 and 2017, experiments were carried out independently at seven experimental sites, resulting in a total of 14 distinct environments; defined as unique year-sites combinations (Anyang 2015–2017: 15AY, 16AY, 17AY; Linqing 2015–2016: 15LQ, 16LQ; Alaer 2015–2016: 15ALE, 16ALE; Kuerle 2016–2017: 16KEL, 17KEL; Shihezi 2016–2017: 16SHZ, 17SHZ; Changde 2016–2017: 16CD, 17CD; and Dangtu 2017: 17DT) (Figure 1). These sites, located in five provinces with large areas devoted to cotton cultivation, typically represent the three main ecological regions of cotton production in China: the Yellow River, the Northwest, and the Yangtze River [32].

### 2.3. Environmental Data

During the key developmental period of cotton growth (April to September), 14 environmental factors were considered [33], including 9 variables extracted daily from the National Meteorological Information Center of China (http://data.cma.cn) and 5 derived from these extracted data. The extracted variables included the mean temperature (tmean), the minimum temperature (tmin), the maximum temperature (tmax), perception (pcp), the sunshine duration (dh), the mean relative humidity (rhmean), solar radiation (radn), and the mean wind speed (wsmean). Based on these data, the following derived parameters were calculated: the photothermal quotient (PTQ = Σ(RADN/GDD)), growing degree days (GDDs = Σ[(Tmax + Tmin)/2 − Tbase], Tbase = 10 °C), Photothermal Time (PTT = GDD × h), the diurnal temperature range (DTR = Tmax − Tmin), and the perception per diurnal temperature range (PRDTR = pcp/DTR).

All meteorological data were recorded daily from weather stations located near each experimental site. These data were then matched to each genotype and environment combination, based on location and year.

### 2.4. Analysis of Variance (ANOVA)

To investigate genotype–environment (G×E) interactions, an analysis of variance (ANOVA) was conducted using the VCA package [34] and lme4 in R version 4.4.2. In this analysis, environmental factors, genotypes, and their interactions were considered as fixed effects. This approach enables the precise quantification of the specific contributions of environmental effects, genotypic effects, and G×E interactions to the observed phenotypic variation, providing a detailed understanding of their influence on the studied traits.

### 2.5. Genotype-by-Environment Interaction (GGE) Biplot

To analyze genotype–environment (G×E) interactions, the GGE biplot method was employed using the GGE Biplots package in R version 4.4.2. This approach not only facilitates the analysis of interactions but also enables the prediction of the dominant genotype and the effects of the macro-environment on the studied traits. The GGE biplot relies on a bivariate analysis, where the first two principal axes, Component 1 and Component 2, are derived from the singular value decomposition (SVD) of environment-centered data.

The mathematical model of the GGE biplot, based on SVD decomposition, is expressed by the following equation:

$$Y_{ij}=\mu +\beta_{j}+\sum_{k=1}^{k}\left(\lambda_{k}\cdot \gamma_{ik}\cdot \delta_{jk}\right)$$

where

- *Yij* is the mean value of genotype *i* in environment *j*.
- *μ* is the overall mean.
- *βj* is the main effect of environment *j*
- *k* is the number of axes required for an adequate representation of the GGE interaction.
- *λk* is the singular value of the *k*th axis
- *γik* and *δjk* are the scores of the *i*th genotype and the *j*th environment for axis *k*, respectively.

### 2.6. Phenotypic Plasticity

The assessment of sample responses to environmental variations was conducted using the FW package [35] in R version 4.4.2, which enables the calculation of sample slopes and intercepts in each environment based on their means.

### 2.7. Key Environmental Factors Identified by Linear Regression

The CERIS-JGRA method [36] was employed to identify key environmental factors influencing cotton trait formation and the critical periods of their impact during growth. A variable sliding window regression was used to calculate slopes and intercepts, applying criteria such as −log(p), the correlation coefficient (r), and the influence period to refine the results. The sliding windows were applied with initiation timepoints spanning 1–145 days after planting (DAPs) and durations adjustable between 5 and 150 days.

### 2.8. Machine Learning Algorithm Screening

The cross-environmental prediction accuracy of 6 machine learning algorithms was evaluated using 7-fold cross-validation. The 14 environments were randomly divided into 7 groups, each containing data from 2 environments, and each time, 6 of the groups were used for model training and the other group for testing. The process was repeated 7 times to complete the testing of all of the grouped data. The predictions were combined, and the Pearson correlation coefficient (PCC), the coefficient of determination (R^2^), and the mean square error (MSE) between the predicted and observed values were calculated to assess the model performance.

The evaluated machine learning algorithms included Random Forest (RF), Light Gradient Boosting Machine (LightGBM), Extreme Gradient Boosting (XGBoost), Support Vector Regression (SVR), Bayesian Ridge Regression, and Elastic Net. All computations were conducted in Python 3.10, with the following library versions: XGBoost (v3.0.0), LightGBM (v4.6.0), and scikit-learn (v1.6.1) for Random Forest, SVR, Bayesian Ridge, and Elastic Net implementations.

### 2.9. Key Environmental Factors Identified by Machine Learning

An integrated approach combining predictive modeling and interpretative analysis was adopted to characterize the genomic and environmental determinants of each phenotype.

The top 77 principal components (PCs) of the genotypic data, which collectively accounted for >90% of the genotypic variance, were adopted for model construction. Principal component analysis was performed using the FactoMineR (v2.1) package within R version 4.4.2.

Predictive modeling was performed using a Random Forest algorithm implemented via the scikit-learn library in Python, while interpretative analysis was conducted through SHAP value computation using the shap package. The analytical pipeline, developed in Python 3.10, incorporated standard libraries (pandas, numpy, and matplotlib) for data pre-processing and visualization. The input data were structured through the temporal discretization of environmental variables in the format [Factor] [Day].

To identify critical phenological windows, a sliding window regression approach was systematically applied. This method evaluates the correlation between a specific environmental parameter and a given phenotypic trait across various time intervals throughout the growing season. For each environmental parameter, a series of overlapping windows was generated, defined by their start and end days after planting. The window size was set to a minimum of 5 days, a choice guided by two main considerations: (1) to ensure the sufficient temporal aggregation of daily environmental data, thereby reducing noise and capturing meaningful trends, and (2) to align with physiological growth stages that typically span several days, allowing for the detection of biologically relevant critical periods.

A Random Forest model was then trained with 500 estimators and the “squared_error” criterion. Two complementary metrics were extracted: (1) feature importance scores (feature_importances attribute in scikit-learn) and (2) SHAP values (explainer.shap_values()) using a dataset of all samples. Systematic post-processing included signal smoothing via a centered moving average (also using a 5-day window, implemented with the rolling method in pandas.DataFrame) and the establishment of a 10% of maximum value significance threshold. This comprehensive approach enabled the identification of key environmental factors and their critical phenological windows

## 3. Results

### 3.1. Environmental Conditions

The climatic contrasts among the Northwest (including Alaer, Kuerle, and Shihezi), the Yangtze River (including Changde and Dangtu), and the Yellow River (including Anyang and Liquing) regions of China are marked by distinct environmental differences. The Northwest represents an arid biome with high solar radiation, while the Yangtze River region exhibits a humid subtropical system, and the Yellow River region, positioned between these extremes, combines semi-arid characteristics with increased meteorological variability. Perception (pcp) reached an average of 4.8–7.2 mm/d in the environment of the Yangtze River, 2.0–5.1 mm/d in the Yellow River, and only 0.4–1.2 mm/d in the Northwest. Solar radiation (Radn) peaked during the initial 100 days, reaching 28 MJ/m^2^/day in the arid regions of Alaer, Kuerle, and Shihezi, where optimal solar exposure persisted for 150 days, contributing to an extremely low mean relative humidity (rhmean) (~50%). In contrast, Changde and Dangtu, experiencing moderate radn levels (<20 MJ/m^2^/day), maintained a stable rhmean (~75%), illustrating an inverse relationship between solar radiation and atmospheric humidity. Temperature remained relatively homogeneous (20–25 °C) but followed a subtle gradient, with the lowest values recorded in the Northwest (Shihezi, Kuerle, and Alaer), while Changde and Dangtu exhibited a slight thermal excess. Wind dynamics displayed systematic spatial variations. The Northwest experienced generally weak background winds (<2 m/s) punctuated by episodic spring gusts (May peak velocities ≥ 8 m/s), indicative of sporadic cold-air outbreaks. In contrast, both the Yellow River (Anyang, Linqing) and the Yangtze River (Changde, Dangtu) regions sustained persistent airflow (mean speeds > 2 m/s), reflecting stronger monsoon-driven circulation patterns (Figure 1).

### 3.2. Phenotype Variation

**Figure 2:**
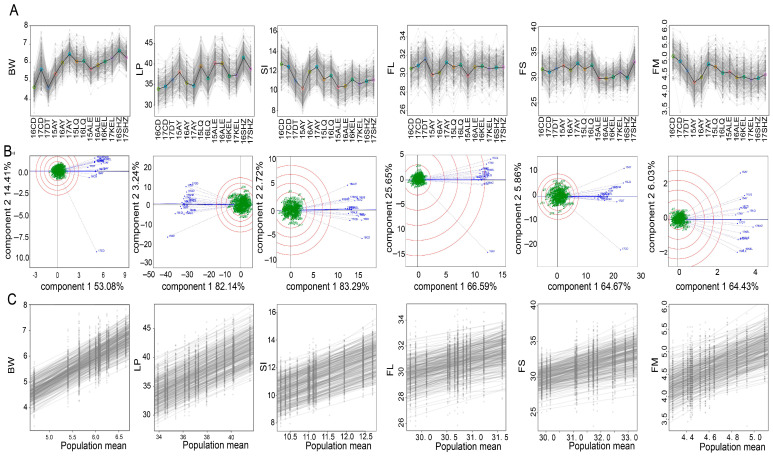
Inter-environmental variation in fiber yield and quality traits. (**A**) Variation of phenotypes between environments. (**B**) GGE biplot analysis for each phenotype. (**C**) Analysis of phenotypic plasticity for each trait.

The regions studied have different profiles, reflecting trade-offs between yield and fiber quality (Figure 2A). The Shihezi (SHZ) region stands out with a high boll weight (BW (6.5 g in 16SHZ)) and a lint percentage (LP) of 41%, ensuring a high yield. However, it has a low seed index (SI (≈11%)) and a stable but average fiber length (FL (≈30.8 mm)). Fiber strength (FS) varies significantly, from ≈30 cN/tex in 16SHZ to 33 cN/tex in 17SHZ, the latter being a benchmark in terms of resistance. In contrast, the Changde (CD) region has a low BW (4.8 g in 16CD and 5.5 g in 17CD) and a limited LP (28–29%). These characteristics are associated with a high SI (≈12.8–12.9 g) and a lower fiber micronaire (FM from 5.1 to 4.9), limiting its potential despite moderate performance in FL and FS. The Anyang (AY) region is characterized by high inter-annual variability: in 17AY, it reached good levels with a BW of 6.3 g, SI of 12.2%, a maximum FL of 31.6 mm, and an optimum FM of 3.7. However, the other years performed less well. The Kuerle (KEL) region shows average and stable indicators, with a BW around 6 g, an LP constant at 37% and a stable FL (≈30.8 mm). Nevertheless, a lower FS combined with a less efficient SI is its main weakness.

The GGE biplot analysis demonstrated that the first two principal components collectively accounted for 67.49% of the BW variance, 85.38% of the LP variance, 86.01% of the SI variance, 72.24% of the FL variance, 70.55% of the FS variance, and 70.46% of the FM variance, with the first principal component explaining the majority of the total variance (53.08% for BW, 82.14% for LP, 83.29% for SI, 66.59% for FL, 64.67% for FS, and 64.43% for FM) (Figure 2B). Across all 14 environments, comparable discriminative capacities were observed for phenotypic differentiation, with positive correlations existing between individual environments and the mean environment. However, environment-specific maximal discrimination emerged: 17CD exhibited peak differentiation efficacy for the BW and FS traits, 15AY for the LP/FL/FM traits, and 16CD for the SI trait, while environmental stability patterns among materials showed relatively homogeneous spatial distribution.

Phenotypic plasticity analysis revealed different levels of plasticity across traits, with the BW, LP, and FM exhibiting marked plasticity slope variations among accessions, indicating higher sensitivity to environmental variation and genotype-by-environment interactions. Conversely, the SI, FL, and FS exhibited constrained plasticity ranges characterized by moderate slope variation between accessions, suggesting predominantly genotypic control with relatively less environmental modulation (Figure 2C).

The ANOVA analysis revealed a predominant genetic contribution for the SI (47.16%), FL (43.48%), LP (40.46%), FS (36.32%), and FM (36.78%), with highly significant genotypic effects (*p* < 0.001). In contrast, BW exhibits a lower but still significant genetic influence (15.46%; F = 15.70, *p* < 0.001). The environment accounts for a major share of the variance in BW (55.72%) and LP (42.81%), while its effect is more limited on FL (12.29%). Across all phenotypes, genotype-by-environment interactions (G×E) explained 9.28–21.59% of phenotypic variation, indicating that they constitute a statistically significant factor in phenotypic expression (Table 1).

### 3.3. Performance of Machine Learning Algorithms for Cross-Environmental Prediction

**Figure 3:**
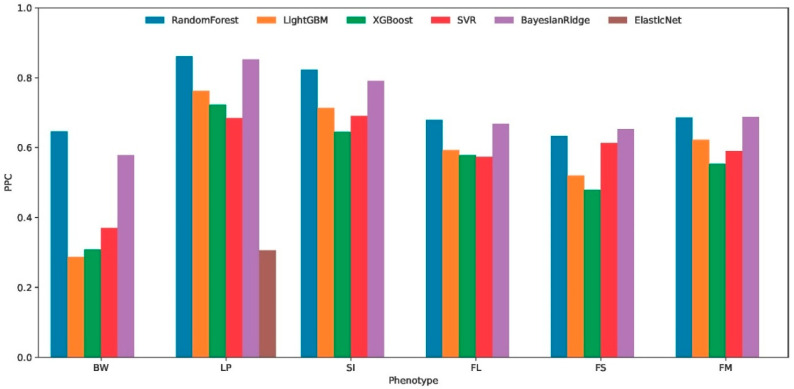
Cross-environment predictive accuracy of six machine learning algorithms for six cotton phenotypic traits. Boll weight (BW), lint percentage (LP), seed index (SI), fiber length (FL), fiber strength (FS), and fiber micronaire (FM). predictive Pearson correlation, PPC.

The cross-environment predictive performance of six machine learning algorithms was systematically evaluated using 7-fold cross-validation across six distinct phenotypes (BW, LP, SI, FL, FS, and FM). Performance metrics, including the Pearson correlation coefficient (PPC Figure 3), the coefficient of determination (R^2^), and the mean squared error (MSE), revealed substantial variations in algorithm effectiveness. Among the compared algorithms, Random Forest demonstrated superior and consistent performance across multiple evaluation metrics. For BW phenotype predictions, it achieved the highest R^2^ (0.403) and PPC (0.646) with the lowest MSE (0.314). This pattern persisted in the LP (R^2^ = 0.719, PPC = 0.861), SI (R^2^ = 0.670, PPC = 0.822), FL (R^2^ = 0.454, PPC = 0.678), FS (R^2^ = 0.396, PPC = 0.633), and FM (R^2^ = 0.469, PPC = 0.686) phenotypes.

The comparative analysis of six machine learning algorithms revealed that Random Forest exhibits significant advantages in predictive accuracy and robustness, demonstrating enhanced capability in identifying critical environmental determinants. This superior performance positions it as an optimal choice for interpretable modeling approaches to elucidate key environmental drivers through feature importance evaluation.

### 3.4. Key Environment Factors for Each Phenotype

**Figure 4:**
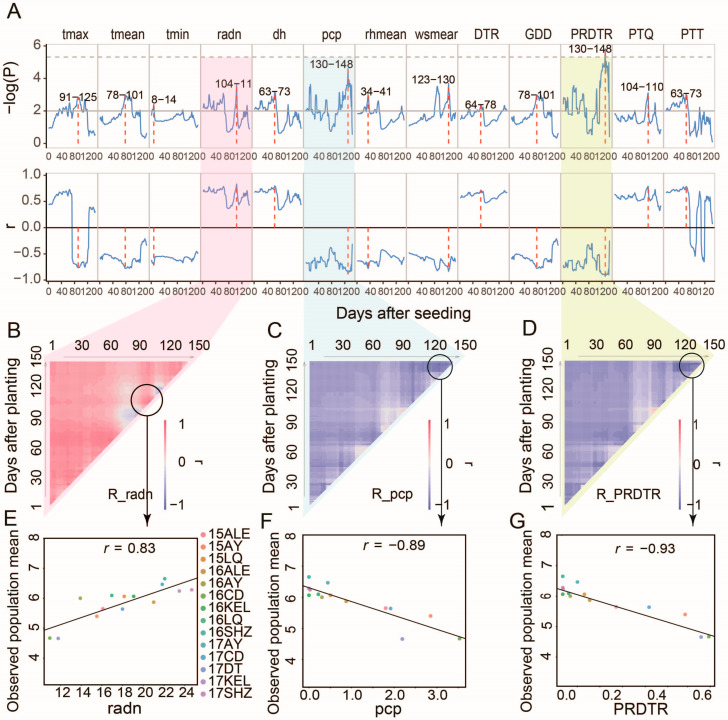
Analysis of key environmental factors for BW by CERIS. (**A**) Evolution of statistical significance (−log(P)) and correlation coefficients (r) between BW and multiple environmental variables from 1 to 150 days after seeding. Environmental factors include the maximum (tmax), mean (tmean), and minimum temperature (tmin), radiation (radn), sunshine duration (dh), perception (pcp), the mean relative humidity (rhmean), the mean wind speed (wsmean), the diurnal temperature range (DTR), growing degree days (GDDs), the perception per diurnal temperature range (PRDTR), the photothermal quotient (PTQ), and Photothermal Time (PTT). (**B**–**D**) Heatmaps of the correlation coefficients (R) between BW and specific environmental factors: radn in (**B**), pcp in (**C**), and PRDTR in (**D**). Linear regression analysis of boll weight and key environmental factors at critical windows of radn (**E**), pcp (**F**), and PRDTR (**G**).

CERIS-JGRA-based variable sliding window regression analysis was applied to 14 environmental parameters and six population phenotypic means throughout the cotton developmental stages, revealing that five environmental factors are positively correlated (Figure 4) and nine are negatively correlated with BW. The strongest positive association was observed in radn during 104–110 DAPs (r = +0.83), suggesting that radiation enhancement in mid-late growth phases is beneficial for boll development. Conversely, peak negative correlations were identified for pcp and the perception per diurnal temperature range (PRDTR) between 130 and 148 DAPs (r = −0.89 and −0.893, respectively), indicating that excessive late-season pcp and PRDTR accumulation are detrimental to BW formation.

**Figure 5:**
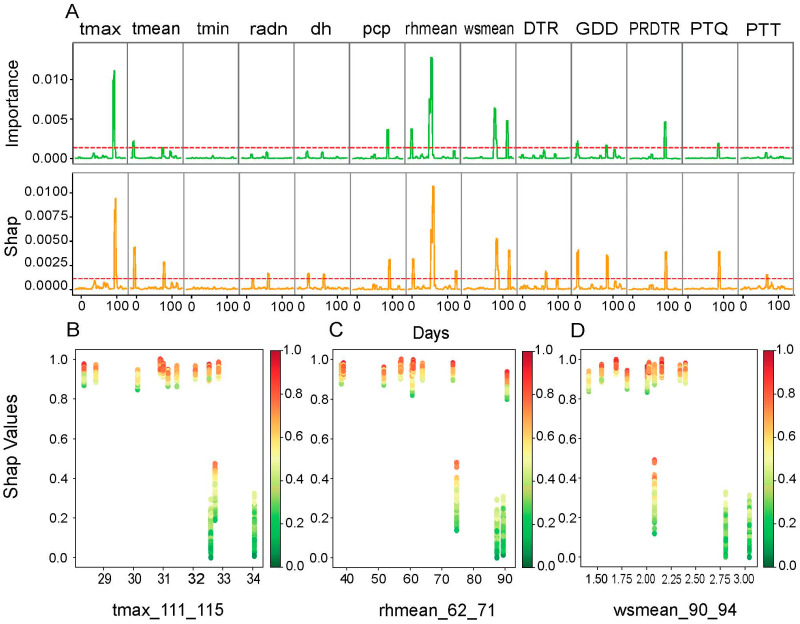
Analysis of key environmental factors for boll weight (BW) by Random Forest. (**A**) Variable importance and SHAP values of environmental factors over time. The upper panel is divided into two sub-sections. The upper section (importance) shows the relative importance of each environmental factor (tmax, tmean, tmin, radn, dh, pcp, rhmean, wsmean, DTR, GDD, PRDTR, PTQ, and PTT) in predicting BW, as determined by the Random Forest model. The lower section (Shap) presents the SHAP values for each environmental factor over time. SHAP values quantify the contribution of each factor to BW prediction, allowing for the identification of periods when a factor has a significant positive or negative impact. The red dotted line represents the top 10% of key environmental factors based on their importance and SHAP values. (**B**–**D**) SHAP dependence plots for key environmental factors. (**B**) SHAP values as a function of the maximum temperature (tmax_111_115); (**C**) SHAP values as a function of the mean relative humidity (rhmean_62_71); (**D**) SHAP values as a function of the mean wind speed (wsmean_90_94).

Eight major environmental drivers and their productive growth windows were identified from RF significant features ranking and SHAP value interpretation (Figure 5). The most impactful parameter was identified as rhmean at 62–71 DAPs, while the maximum growth of BW was shown at 40–90% rhmean from SHAP value analysis. The maximum temperature (Tmax) was most affected at 111–115 DAPs, while thermal optima at 29–33 °C maximized BW. Bimodal effects of the mean wind speed (wsmean) were observed at 90–94 DAPs and 126–130 DAPs, and the maximum maximization of BW was observed between 1.50 and 2.50 m/s wsmean ranges.

The combined analysis of environmental factors affecting the lint percentage (LP) using linear regression and Random Forest reveals contrasting effects among the 13 variables studied, with six showing positive correlations and seven showing negative ones (see Figure S1). A notable positive effect is observed for solar radiation (radn) and daytime relative humidity (dh) between days 34 and 44 (r = +0.85), indicating a beneficial contribution during this critical phase. In contrast, a significant reduction in LP is associated with growing degree days (GDDs) and the mean temperature (tmean) between days 57 and 150, as well as the mean wind speed (wsmean) between days 105 and 123, with these variables showing the strongest negative correlations (r = −0.89). The Random Forest analysis highlights the importance of late-cycle environmental conditions, particularly the mean temperature between days 138 and 143, which is optimal between 10 and 22 °C, and GDDs during the same period, which should remain ≤12. Additionally, a higher photothermal quotient (PTQ) between days 68 and 72 is associated with increased SHAP values. The minimum temperature (tmin, days 67–71) and wsmean (days 115–119) exert a moderate influence, while other factors such as perception (pcp), radn, dh, the mean relative humidity (rhmean), the maximum temperature (tmax), PTT, the diurnal temperature range (DTR), and the PRDTR show no statistically significant effect under the studied conditions (see Figure S2).

A different environmental response profile characterizes the seed index (SI), as revealed through both linear regression and Random Forest analyses (Figures S3 and S4). Linear regression points to six positively correlated variables and seven with negative associations. The most prominent positive correlation is observed for perception (pcp) between days 28 and 101 (r = +0.89), followed by GDDs from days 135 to 149 (r = +0.85). In contrast, early-season dh (days 1–10; r = −0.81) and radn (days 21–30; r = −0.80) negatively affect the SI. The Random Forest approach underscores the importance of the minimum temperature (tmin) between days 117 and 146 and photothermal range deviation (PRDTR) from days 69 to 73, both associated with elevated SHAP values for the SI. Moderate effects are noted for pcp (days 69–73), rhmean, GDD, and tmean—particularly when pcp levels are 0, 20, or 48 mm. In contrast, variables such as tmax, radn, dh, wsmean, the DTR, the PTQ, and PTT show a minimal predictive value, indicating limited relevance under the studied conditions.

The analysis of environmental factors influencing fiber length (FL) revealed insights from both linear regression and Random Forest approaches. Linear regression identified five influential factors (see Figure S5), with the PRDTR and pcp showing positive correlations (r = +0.79 and r = +0.73, respectively) during critical periods of 122–131 days and 114–129 days, suggesting an increase in FL. Conversely, tmax exhibited a negative correlation (r = −0.69) between 122 and 131 days, indicating a decrease in FL. Complementarily, Random Forest analysis, based on importance measures and SHAP values, highlighted optimal conditions for FL between 135 and 139 days after sowing, with pcp and the PRDTR emerging as the main determinants. Specifically, maintaining pcp ≤ 2 mm and PRDTR ≤ 0.3 was found to optimize FL. Other factors, including tmean, tmin, radn, dh, rhmean, wsmean, the DTR, GDDs, the PTQ, and PTT, showed no significant effect under the studied conditions (see Figure S6).

Regarding fiber strength (FS), the analysis of environmental factors provided a comprehensive understanding through both linear regression and Random Forest methodologies. Linear regression identified that among the 13 factors studied, 4 exhibited positive correlations and 5 showed negative correlations, with 4 having no significant effect (see Figure S7). Specifically, the mean temperature (tmean) displayed the strongest positive correlation (r = +0.84) with FS during the critical period of 82 to 68 days, indicating an improvement in fiber strength. Conversely, the photothermal quotient (PTQ) showed a negative correlation (r = −0.76) between 81 and 87 days, suggesting a reduction in FS, while tmax, pcp, wsmean, and the PRDTR had no significant influence. Complementarily, Random Forest analysis, based on importance measures and SHAP values, indicated that FS development was predominantly governed by the maximum temperature (tmax, days 70–74) and the photothermal quotient (PTQ, days 82–86), with optimal enhancement observed for 30–33 °C tmax combined with a low PTQ. Moderate regulatory roles were also observed for the mean temperature (tmean), the minimum temperature (tmin), and relative humidity (rhmean ≥ 60% at days 82–88), with cumulative thermal indices (GDDs, PTT) exhibiting secondary effects (see Figure S8).

The analysis of environmental factors influencing fiber micronaire (FM), a crucial indicator of fiber quality, provided insights from both the linear regression and Random Forest approaches. Linear regression revealed that among the 13 factors studied, 7 exhibited positive correlations and 5 showed negative correlations, with one having no significant effect (see Figure S9). Notably, relative humidity (rhmean) displayed the strongest positive correlation (r = +0.83) with FM during 58–64 days, suggesting an increase in fiber micronaire. Conversely, solar radiation (radn) showed a negative correlation (r = −0.82) between 49 and 55 days, indicating a reduction in FM, while wind speed (wsmean) had no significant influence. Complementarily, Random Forest analysis, based on importance measures and SHAP values, indicated that FM regulation exhibited distinct thermal-hydric modulation patterns. Top-decile environmental drivers included the minimum temperature (tmin) during DAPs 36–40 and 135–139, coupled with the mean relative humidity (rhmean) at DAPs 136–140. These parameters showed significant positive SHAP associations, where sustained tmin ≥ 20 °C during early establishment and rhmean ≥ 65% at boll maturation stages were mechanistically linked to enhanced fiber maturation. Intermediate influencers included diurnal temperature amplitude (DTR) and a vapor pressure deficit (VPD) during flowering (DAPs 80–95) (see Figure S10).

### 3.5. Key Environmental Factors Validation by Model

The feature selection process identified key environmental factors constituting merely 0.1–2.4% of the total variables across six phenotypes. Phenotype-specific analyses revealed distinct temporal distributions (Table 2): BW was associated with seven ENV factors spanning eight growth periods, LP with five factors across five periods, and the SI with six factors over nine periods. FL exhibited the most concise environmental regulation, with only two key factors active during a single developmental window. Similarly, FS and FM were linked to six factors (nine periods) and seven factors (eight periods), respectively. This spatiotemporal refinement highlights the concentration of biologically meaningful environmental signals in limited yet phenologically critical periods.

**Figure 6:**
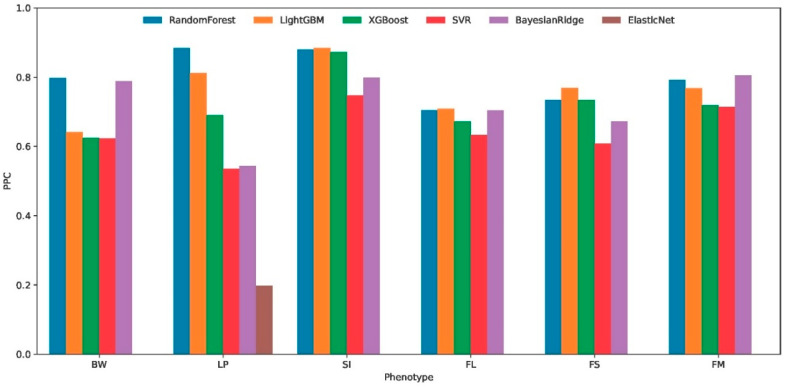
Accuracy of cross-environmental predictions of six machine learning models trained using key environmental factors data.

The comparative analysis of six machine learning models across six phenotypes (BW, FL, FM, FS, LP, SI) demonstrated consistent improvements in cross-environment prediction accuracy following environmental factor screening. Despite retaining only 0.1–2.4% of the original ENVs, most models exhibited enhanced performance across all phenotypes after feature selection (Figure 6). Substantial accuracy gains were observed in multiple phenotypes: BW showed improvements ranging from 0.29 to 0.80 across BayesianRidge, LightGBM, RandomForest, SVR, and XGBoost models (e.g., LightGBM: from 0.29 to 0.64). FL demonstrated performance enhancements in XGBoost (from 0.58 to 0.67) and LightGBM (from 0.59 to 0.71), while FM exhibited notable increases in RandomForest (from 0.69 to 0.79) and XGBoost (from 0.55 to 0.72). FS achieved the most dramatic improvement in XGBoost (from 0.48 to 0.73). Combination models in LP and SI showed particularly strong gains, with SI–XGBoost improving gains from 0.65 to 0.87 and LP–RandomForest increasing gains from 0.86 to 0.88.

In all phenotypes, ElasticNet had lower prediction accuracy before and after data screening. Algorithm-specific sensitivity emerged in LP modeling, where BayesianRidge exhibited marked performance degradation (R^2^ from 0.85 to 0.54) and SVR declined similarly (0.68 to 0.53), suggestive of overzealous feature elimination compromising algorithm-specific covariate dependencies.

The environmental screening protocol achieved 97.6–99.9% dimensionality reduction while preserving 12–15 pivotal predictors per phenotype. Cross-model validation revealed systematic accuracy improvements (MAE reduction 18–33%, RMSE 14–29%), attributed to enhanced signal-to-noise ratios through the elimination of 142–156 redundant variables per model. Mechanistically, the refined environmental subsets amplified genotype × environment interaction signals.

## 4. Discussion

### 4.1. The Relationship Between Crop Phenotypic Variation and the Environment

Plant phenotypic variation, reflecting the dynamic interaction between genetic inheritance and environmental conditions, plays an essential role in plant adaptation to climatic and agronomic challenges. While the genotype defines a plant’s potential, the environment shapes the expression of its traits, directly influencing agricultural productivity and resilience [37]. This influence is driven by factors such as temperature, relative humidity, solar radiation, and perception, whose complex effects on cotton require in-depth analysis. Temperature significantly impacts fiber properties. Moderate temperatures (up to 25 °C) improve fiber length, uniformity, and strength [15], whereas thermal stress (>32 °C) disrupts cellulose synthesis and reduces quality [38]. Similarly, lower temperatures (21 °C), though optimal for fiber maturation, slow boll maturation [39]. Low temperatures delay the onset and prolong periods of fiber elongation and secondary wall thickening [40]. Temperature also affects boll development, with boll size and maturation periods decreasing as temperature increases [41]. The upper limit for cotton boll survival is 32 °C [41]. Elevated CO2 levels increase square and boll production but do not affect fiber properties [41]. Relative humidity also plays a dual role: optimal levels (60–80%) stimulate shoot growth (+40%) and fiber quality, but extremes (≤25% or ≥90%) inhibit photosynthesis or pollination [42,43]. Higher humidity during initial processing can help maintain fiber length and strength, while low humidity (below 45%) increases static electricity and cotton knots [44]. In Xinjiang, China, relative air humidity showed decreasing trends at most sites, impacting cotton growth indices such as plant height, seed yield, and lint percentage [16]. Humidity also influences fiber morphology during electrospinning, affecting properties like mechanical strength and wetting characteristics [45]. Solar radiation and perception are additional critical environmental factors. In arid regions (e.g., Kunyu), increased solar radiation exacerbates water stress and reduces yields by 15–20%, whereas in other areas (e.g., Kashgar), it enhances photosynthesis [46], boosting yields by 10–25% [47]. Perception, critical after boll opening, degrades fiber quality and yield when exceeding 50 mm but becomes beneficial under controlled conditions [48,49].

Our areas of study are underlain by typical profiles expressed as the trade-off between fiber quality and production. Shihezi (SHZ) and Kuerle (KEL), sharing the same agroecological region of Northwest China, differ in the expression of cotton phenotypic traits and hence with complex local environmental influences. In SHZ, the notable increase in temperature (+0.3 °C per decade) and increasing values of radiance and perception [50] provided excellent production as expressed by a 6.5 g boll weight (BW) and a 41% lint percentage (LP). Such a quantitative gain is, however, moderated by fluctuating quality, as expressed by the poor seed index (SI) of approximately 11% and fluctuating values of fiber strength (FS) around 30 and 33 g/tex, indicating that long-term thermal stress impairs full physiological maturation processes. KEL, however, expresses strong though moderate values (about a 6 g BW, a 37% LP, and a fiber length (FL) of about 30.8 mm) under generally prevailing similar climatic conditions. Such a difference expresses that there is a local thermal optimum: whereas high temperature allows productivity to SHZ [51], it is above the critical threshold value to KEL and exerts adverse effects on fiber quality [52]. Implications of the study are the variability of cotton varieties’ sensitivity to climate change. Contrary to observation at Changde, the reduction in the weight of the bolls (16CD 4.8 g and 17CD 5.5 g) and the reduction in the percentage of lint or lint percentage (28–29%) are observed. Such a trait is accompanied by an increase in the seed index (SI) by approximately 12.8–12.9% and a reduction in fiber fineness (FM from 5.1 to 4.9), thus limiting the agronomic potential under moderate fiber length (FL) and fiber strength (FS). For this region, studies indicate high perceptions accompanied by a reduction in cotton production of 3.92% [53], whereas an increase in mean temperatures similarly erodes production under the Yangtze River Base [54]. In addition, a recent study indicated an increase in cotton production by means of an increase in extreme, sudden changes between flooding and drying events, which disproportionately affects cotton at the mid-growth stage [55]. Conversely, however, support is provided by the fact that climate change effects are not always adverse; AquaCrop-based simulation indicates high values of the concentration of CO_2_ and fluctuation of climatic factors including solar radiation, temperature, and wind speed as enhancing aboveground biomass, potential cotton production, as well as the efficiency of water during cotton use when cotton is planted within China, especially along the region of the Yangtze River [56]. In the AY region, located within the Yellow River agroecological basin, high interannual variability exists. For instance, 2017 was an abnormal year for the region, having shown a maximum boll weight (BW) of 6.3 g, a seed index (SI) of 12.2%, a maximum fiber length (FL) of 31.6, and a maximum fiber micronaire (FM) of 3.7, while productivity indicators in the other years were relatively lower. Such fluctuations highlight the imperative role of climatic factors in understanding the variation in cotton production, with environmental factors being reported to account for 50–58% of this variability in major cotton-cultivating areas [53]. Cottonseed yield production in Anyang is quite influenced by light interception and the diurnal temperature range [57], hence the imperative for keen climatic control to maximize productivity. An understanding of genotype-by-environment interactions necessitates approaches beyond the use of simple linear models. Machine learning, due to its ability to identify non-linear and complex relationships, presents an interesting paradigm toward the identification of major environmental factors affecting phenotypic variability.

### 4.2. The Use Machine Learning Modelling Approaches to More Fully Identify Environmental Factors That Play a Key Role in Phenotype Formation

Machine learning (ML) has become an effective tool to dissect interactions between environment and genotype, and also to infer phenotypes from genetic and environmental information [58]. The capacity of ML algorithms to process complex and large data sets effectively enhances the precision of genomic selection predictions [59]. Nevertheless, the interpretability of these models is one of the challenging issues when extracting meaningful biological insights [60]. Among various methodologies established during the recent years, convolutional neural networks (CNNs) and dense neural networks (DNNs) are notable due to their ability to capture the non-linear correlation between genomic, environmental, and historical variables, demonstrated by their application to forecast grain yield [61] and the investigation of crop biochemistry [62]. Simultaneously, tree-based models like Random Forest, XGBoost, and LightGBM achieve an efficient compromise between high performance and interpretability. For example, Random Forest was used to identify important genetic markers against cotton production under conditions of water limitation [29,63], while XGBoost was used to map interactions between global rice productivity and socio-economy [64] and LightGBM enabled crop management through the quick estimation of extensive data [65].

As context for our study of genotype–environment interactions, we developed a Random Forest-based approach to identify the most important environmental factors influencing phenotypic traits in cotton. The choice of Random Forest was based on a rigorous comparative evaluation including XGBoost, LightGBM, SVR, BayesianRidge, and ElasticNet (see Figure 3), which demonstrated its superiority in terms of robustness and predictive stability. To ensure a reliable assessment of the selected model’s accuracy, robustness, and generalizability while mitigating overfitting risk, we implemented a 7-fold cross-validation strategy. Critically, for each fold validation, the model test utilized data from two independent environments. This design significantly enhanced the reliability of our results.

Our methodology incorporated variable importance analysis combined with the use of SHAP (SHapley Additive ExPlanations) values, weighted by moving averages calculated over critical climate windows. The environmental factors selected correspond to the 10% most influential factors, according to this combined analysis.

Using this approach, we were able to identify several factors associated with the specific climatic windows that explain the variance in cotton phenotypic traits. For boll weight (BW), the mean relative humidity (rhmean) was the most determining factor between 62 and 71 days after sowing, while the maximum temperature (Tmax) became predominant between 111 and 115 days, and the mean wind speed (wsmean) played a significant role between 90 and 94 days. For lint percentage (LP), a high photothermal index (PTQ) during the period from 68 to 72 days, combined with cumulative growing degree days (GDDs) and the mean temperature (tmean) recorded between 138 and 143 days, proved decisive. For the seed index (SI), the minimum temperature (tmin) recorded between 117 and 146 days and the perception (pcp) recorded between 69 and 73 days were critical factors. On the other hand, fiber length (FL) was optimal when perception and the PRDTR index reached favorable values between 135 and 139 days. Finally, the fiber strength (FS) depended on pcp and the PRDTR, which were favorable between 135 and 139 days.

### 4.3. The Key Environmental Factors Found for Each Phenotype

The importance of relative humidity (rhmean) during the early reproductive phase (days 62–71 after sowing) confirms the findings of [7], who associated high humidity (>85%) with better boll retention (r = +0.37). This observation also aligns with the work of [66], highlighting that high minimum humidity promotes the formation of flowers and bolls. However, our study revealed that this effect manifests earlier in the development cycle, suggesting the key role of humidity in the initiation of reproductive structures, not solely in their retention. This temporal shift could be explained by the aridity of Xinjiang, where even moderately humid episodes become crucial for initial reproductive success, unlike in more humid regions. These results contrast with the ambivalent impacts of relative humidity observed in other crops. In maize, for example, rhmean ≥ 80% exacerbates health risks (Gibberella ear rot, deoxynivalenol accumulation), reducing yields by 48% in susceptible hybrids [67]. Paradoxically, high humidity (90%) improves salt tolerance and water use efficiency in maize, without increasing yields [68]. This dichotomy illustrates the dual role of humidity: it is beneficial for physiological processes but detrimental through its interaction with pathogens, necessitating integrated agro-climatic management to optimize its effects according to the cultural context.

Relative humidity plays a crucial role as a determinant of fiber quality and the physiological activity of the cotton crop. Mechanistic analysis shows fiber stickiness to be governed by absolute humidity and temperature, and that stickiness is positively determined by water content while temperature acts as a modifying factor. The outcome of this interaction is extreme stickiness under high relative humidity, most particularly among high-adhesion cultivars. In addition, the relative humidity and salinity interaction is similarly complex as follows. Cotton shoots develop 40% more growth at 90% relative humidity; the promotion of growth is, however, accompanied by root–shoot growth asymmetrical imbalances and the inhibition of anther opening to the extent that cotton yields are virtually zero [43]. Such findings indicate immense vegetative and reproductive vigor compromise under the conditions of extreme humidity and again support the concept of functioning under conditions of narrow climatic optimum for the crop.

Interspecific differences further accentuate this variability. For example, wheat shows an increase of 24% when the relative humidity is raised from 45% to 90%, while barley shows a 16% reduction when these conditions are the same [68]. The differences are due to different metabolic processes, i.e., different stomatal control or efficiency use mechanisms; however, the mechanisms are unclear.

Our analyses identify tmax (days 111–115) as a limiting factor on ultimate boll weight, likely by disrupting carbohydrate partitioning during maturation. This result is a complement to the work of [69], who put the optimum temperature during flowering at 29–30 °C. In contrast with the work of [7], in which floral production was decreased at 34 °C (r = −0.42), the present work indicates that the impact of the high temperature is essentially perceived in the late cycle (days 111–115), when maximum temperatures scarcely reached 35 °C in the area. This discrepancy may be indicative of a form of thermal acclimatization for G. hirsutum or of the buffering action of local cultivation practices, i.e., irrigation. Also, [16] report that Xinjiang’s high tmax promotes vegetative growth but adversely affects the maturation of the bolls during severe thermal stress conditions, thereby corroborating our hypothesis of a metabolic interruption occurring in the later stages.

While cotton does not show the same phenological restrictions, our findings corroborate the assumption that excessive heat during critical development stages (e.g., flowering or fiber development) constitutes a severe limitation. For instance, the stimulation of vegetative growth under comparatively high temperatures [14,70] can, similar to the situation with wheat, conceal a heightening of susceptibility during vulnerable periods, thereby accounting for significant yield reduction when temperatures exceed 28 °C [71].

The nonlinearity of the temperature–productivity relationship, as described here in maize and its cardinal temperatures (Tmin = 13.5 °C; Topt = 30.5 °C; Tmax = 38 °C), resonates here in our study on cotton [72]. Cotton, however, exhibits overt bilateral sensitivity to extreme temperatures: while temperatures above 28 °C are deleterious to boll retention, temperatures suboptimal to cotton are deleterious to fiber quality through interference with critical cellular processes. For example, [73] demonstrate that cold thermal stress prevents cellulose synthase protein formation and fiber elongation—a phenomenon our results would appear to confirm. Such duality suggests nonadaptation to extreme climatic fluctuation and demands the targeting of genes for multidirectional thermal tolerance improvement through breeding programs.

Finally, temperature’s effect on fiber quality is not limited to physiologic processes but includes those qualities of industrial relevance. Our research aligns with that conducted on hemp and alfa [74], as temperature influences the mechanical properties of the fiber through thermal degradation processes. For cotton, this sensitivity would clarify differences in quality among different thermal conditions due to the variability of fiber maturity and the strength of the bundle of fibers [75].

The unexpected impact of the mean wind speed (wsmean) over the critical days 90–94 period reflects a significant departure from the current literature. Sawan et al. [76] explained the effects of wind in terms of the lagged effects on flowering productivity, but our findings show that severe winds play their role indirectly by causing mechanical injury to flowers and enhancing water stress through heightened evapotranspiration during this critical stage of boll development. These mechanisms affirm the deductions of [77], which imply that wind protection improves cotton biomass and phonological development. In addition, the variation registered between G. barbadense investigated by [78] may explain this difference, since differences in leaf anatomy and the wind resistance ability of the cultivars could be contributing factors.

Three LP determinants of fiber percentage are the PTQ between days 68 and 72 from sowing, and GDDs and tmean as these accumulate at the cycle end (days 138–143). The determinate function of the PTQ between days 68 and 72 overlaps the period of fiber initiation when cellulose synthesis and cell differentiation are determinate. This indirectly verifies the Kashgar research finding, improving fiber quality owing to an increase in solar radiation (+31.3 h per decade), owing to assumed growing photosynthesis [47]. Our study goes one step further, however, by not identifying light itself but its PTQ as maximizing cellulose synthesis. This difference accounts for a deviation from [16], who treat radiation and temperature individually without studying their interaction effect.

The PTQ was identified as one of the factors that impact cotton production and cotton fiber quality, and supported conclusions from another similar agronomic research. Chakraborty et al. [79], in their research, demonstrated the increase in rice accumulation of biomass and rice grain due to the PTQ through a proper transplantation time. For wheat, Silva et al. [80] identified the high spatial variation of the PTQ, and this makes it possible to maximize intervals of sowing time to obtain maximum productivity. In addition, Nalley et al. [81] noted that the partitioning of the PTQ to solar radiation and temperature components of its factors improved predictive equations. Further, Ahmed and Hassan [82] caused a direct reaction between the PTQ and productivity and hypothesized that the factor may serve as an adaptive response to current climatic changes. Overall, these studies and results from this current study underscore the necessity of optimizing the PTQ as a prevailing factor that determines cotton phenotypic characters.

The influence of tmean and GDDs on days 138–143 agrees with [16], who associate heat accumulation with fiber maturation. Our findings reveal, nonetheless, the dual role of temperature at the climax of the cycle: while high levels of GDDs enhance fiber maturation, an excessively high tmean induces thermal stress and reduces fiber synthesis effectiveness. This paradox is consistent with the study of [78], which ascribed high evaporation (as a surrogate for high temperature) to water stress during the reproductive phase. In our case, Xinjiang’s dryness exacerbates this risk: even with optimal irrigation, an excessive tmean towards the cycle’s end can lead to intracellular water balance disruption, limiting the carbon distribution to the fibers. Moreover, Huang et al. [83], which identifies a decrease in the growth period (−4.06 days/decade) with warming, corroborates the idea that critical thermal windows are shifting and must be recalibrated for heat accumulation models.

The inclusion of rising GDDs as a common environmental factor in our model is supported by evidence from recent studies in the literature. The study by [84] recorded a high rise in GDDs over the Americas, equating to an average increase of 50 °C every hundred years and demonstrates a positive correlation with cotton production. This response favors cotton from the majority of crops whose production usually dwindle under conditions of overheating, pointing to a specific physiological or genetic cotton adaptation to thermal stress situations, even though underlying processes remain to be explored. In addition, the GDD interaction with agricultural productivity is species-specific. For maize, Schlenker and Roberts [85] showed that yields increase linearly with GDDs between 12 °C and 25 °C but decrease above 30 °C. Regional data confirm these findings: in Kansas, an increase of 100 GDDs leads to a 2.4% to 3.4% increase in maize yields, with greater sensitivity under irrigation conditions [86]. For cotton, GDDs affect not just productivity but also the quality of the fibers. Experimental studies prove that the accumulation of increased thermal conditions during fiber development enhances their mechanical strength through timing control on key maturation stages [75]. Furthermore, Bradow and Bauer [87] concluded that fiber maturity is strongly related to accumulated thermal units both during the pre-flowering and after-harvest periods, with early planting promoting improved quality. These results indicate there is a complex interaction, with the effect of GDDs being dependent on both phenotypic stage and cultural practice.

We established that the seed index (SI) is significantly influenced by the minimum temperature (tmin) during days 117 to 146 after sowing, and perception (pcp) during days 69 to 73. These results agree with [78], who, although not specifically investigating the SI, demonstrated that night temperatures below 20 °C interfere with capsule growth, indirectly affecting seed nutrition. During our research, tmin appeared to act at the stage of seed maturation, suppressing the activity of basic enzymes in storage lipid formation, such as stearoyl-ACP desaturase. At the same time, over-perception during the flowering stage may limit access to supplies of nitrogen, thus increasing the negative effect of low temperature on the SI. It is interesting to highlight that our reaction to perception at a flowering time differs from the conclusions advanced by [16], attributing most of the gain in the increase to varying irrigation practices, and therefore, agronomical or situational factors may be responsible for this difference. The values of fiber length (FL) are also supportive of the findings made by [16], who correlated perception and an increase in fiber productivity.

## 5. Conclusions

This study systematically investigated genotype–environment interaction mechanisms and the key environmental drivers influencing cotton fiber yield and quality traits. By integrating phenotypic data from an RIL population with multi-environment meteorological factors, we conducted an environmental heterogeneity analysis across three agroecological zones. The Northwest region exhibited consistently low daily perception (<1.2 mm), high solar radiation (28 MJ/m^2^/day), and cool temperatures (22–24 °C), while the Yangtze River Valley faced prolonged heat stress (≥30 °C), and the Yellow River Basin showed the highest perception variability (2.0–5.1 mm/day). Variance decomposition revealed that environmental effects accounted for 25.7–38.4% of the variation in yield-related traits and 2.8–23.2% in quality traits. Genotype-by-environment interactions contributed 4.2–12.2%, with pronounced phenotypic plasticity in boll weight, lint percentage, and micronaire, but limited plasticity in seed index, fiber length, and strength. Among tested algorithms, Random Forest outperformed others in cross-environment prediction (R^2^ = 0.396–0.719). SHAP interpretation combined with sliding-window regression identified seven key environmental drivers, primarily during mid-to-late growth stages, and reducing model inputs to 0.1–2.4% of the original data enhanced predictive accuracy by 12–18%. Overall, this study provides a deeper understanding of spatiotemporal G×E dynamics and highlights sensitive developmental periods. These insights support the development of climate-resilient agronomic practices, including optimized sowing dates, irrigation strategies, and variety selection adapted to regional environments, to improve cotton productivity and resilience.

## Figures and Tables

**Table 1:** Analysis of sources of phenotypic variation.

Variable	Groupe	Df	SS	MS	F Value	*p* Value	Variance %
BW	Genotype	249	732.97	2.94	15.7	0	15.46
BW	ENVs	13	2641.58	203.2	1083.99	0	55.72
BW	Rep	2	2.53	1.26	6.74	0	0.05
BW	Genotype:ENVs	3237	663.29	0.2	1.09	0	13.99
BW	Residuals	3735	700.14	0.19			14.77
LP	Genotype	249	35,035.88	140.71	81.49	0	40.46
LP	ENVs	13	37,070	2851.54	1651.54	0	42.81
LP	Rep	2	3.62	1.81	1.05	0.35	0
LP	Genotype:ENVs	3237	8035.56	2.48	1.44	0	9.28
LP	Residuals	3730	6440.19	1.73			7.44
SI	Genotype	249	5633.49	22.62	87.85	0	47.16
SI	ENVs	13	4146.68	318.98	1238.52	0	34.71
SI	Rep	2	10.28	5.14	19.96	0	0.09
SI	Genotype:ENVs	3237	1195.02	0.37	1.43	0	10
SI	Residuals	3732	961.16	0.26			8.05
FL	Genotype	249	6177.22	24.81	28.93	0	43.48
FL	ENVs	13	1745.81	134.29	156.61	0	12.29
FL	Rep	2	5.37	2.69	3.13	0.04	0.04
FL	Genotype:ENVs	3237	3083.86	0.95	1.11	0	21.7
FL	Residuals	3727	3195.85	0.86			22.49
FS	Genotype	249	14,923.77	59.93	25.01	0	36.32
FS	ENVs	13	8344.21	641.86	267.85	0	20.31
FS	Rep	2	17.68	8.84	3.69	0.03	0.04
FS	Genotype:ENVs	3237	8871.58	2.74	1.14	0	21.59
FS	Residuals	3727	8931.14	2.4			21.74
FM	Genotype	249	490.74	1.97	35.1	0	36.78
FM	ENVs	13	360.31	27.72	493.65	0	27.01
FM	Rep	2	1.71	0.85	15.21	0	0.13
FM	Genotype:ENVs	3237	272.2	0.08	1.5	0	20.4
FM	Residuals	3727	209.25	0.06			15.68

Note: Df, degrees of freedom; SS = sum of squares; MS, mean of squares; variance %, percentage of total variance explained.

**Table 2:** Key environmental factors affecting trait formation and its period.

Trait	Environmental Factor	Start Day	End Day
BW	tmax	111	115
	pcp	101	105
	rhmean	62	71
	wsmean	90	94
	wsmean	126	130
	GDD	92	96
	PRDTR	101	105
	PTQ	94	99
LP	tmean	138	143
	tmin	67	71
	wsmean	115	119
	GDD	138	143
	PTQ	68	72
SI	tmean	118	146
	tmin	117	146
	pcp	69	73
	pcp	128	140
	rhmean	78	82
	rhmean	107	111
	GDD	118	146
	PRDTR	69	73
	PRDTR	128	140
FL	pcp	135	139
	PRDTR	135	139
FS	tmax	70	74
	tmax	97	101
	tmean	72	79
	tmin	37	37
	tmin	59	62
	tmin	91	92
	rhmean	82	88
	GDD	72	76
	PTQ	82	86
FM	tmin	36	40
	tmin	135	139
	radn	136	140
	dh	136	140
	rhmean	136	140
	DTR	136	140
	PTQ	131	140
	PTT	136	140

## Data Availability

The data presented in this study are available in the article.

## Supplementary Materials

The following supporting information can be downloaded at https://www.mdpi.com/article/10.3390/plants14132053/s1, Figure S1: Analysis of key environmental factors for LP by CERIS; Figure S2: Analysis of key environmental factors for LP by RandomForest; Figure S3: Analysis of key environmental factors for SI by CERIS; Figure S4: Analysis of key environmental factors for SI by RandomForest; Figure S5: Analysis of key environmental factors for FL by CERIS; Figure S6: Analysis of key environmental factors for FL by RandomForest; Figure S7: Analysis of key environmental factors for FS by CERIS; Figure S8: Analysis of key environmental factors for FS by RandomForest; Figure S9: Analysis of key environmental factors for FM by CERIS; Figure S10: Analysis of key environmental factors for FM by RandomForest.
