# Supplementary figures and images for: Machine Learning-Driven Identification of Key Environmental Factors Influencing Fiber Yield and Quality Traits in Upland Cotton
Source: Plants (Basel). 2025 Jul 4;14(13):2053. doi: 10.3390/plants14132053 (PMC12252131; doi:10.3390/plants14132053)

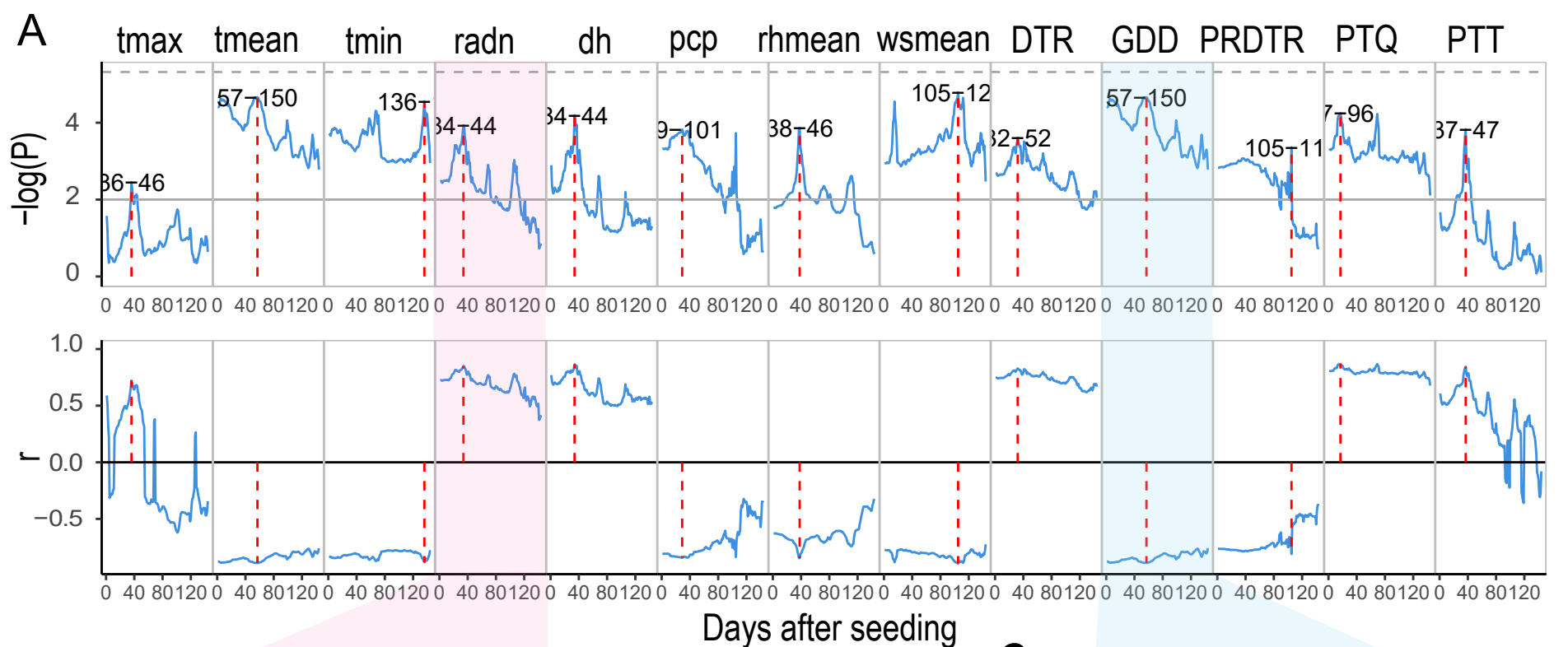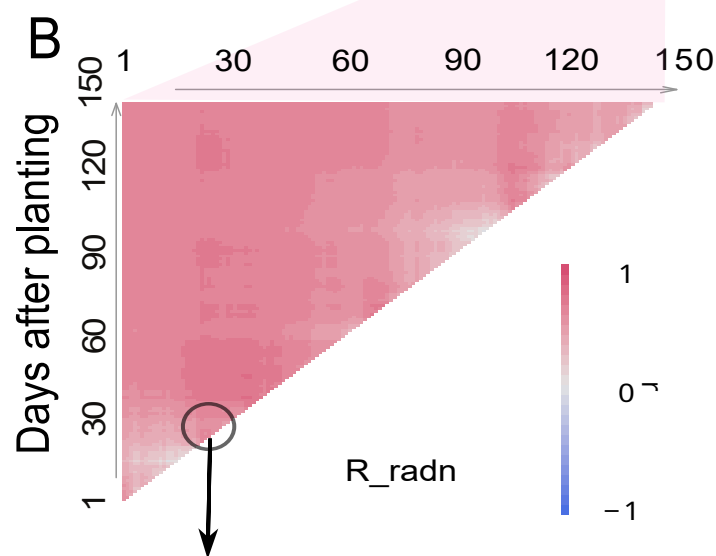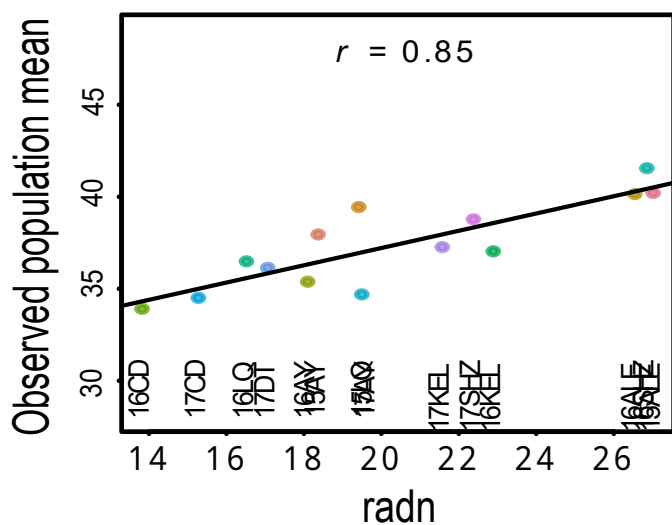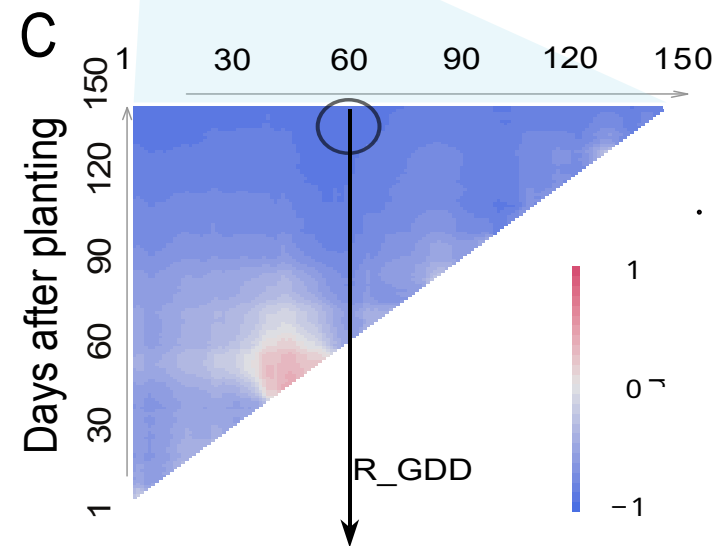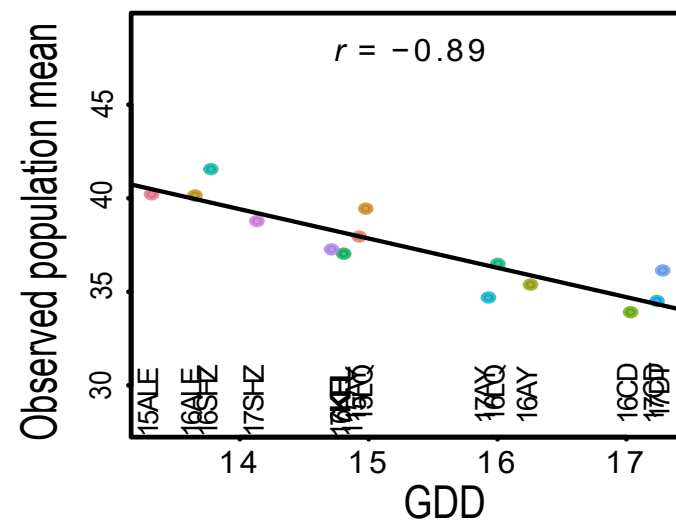

Supplement: Supplementary file 1 [file plants-14-02053-s001.zip › fig_S1.pdf]

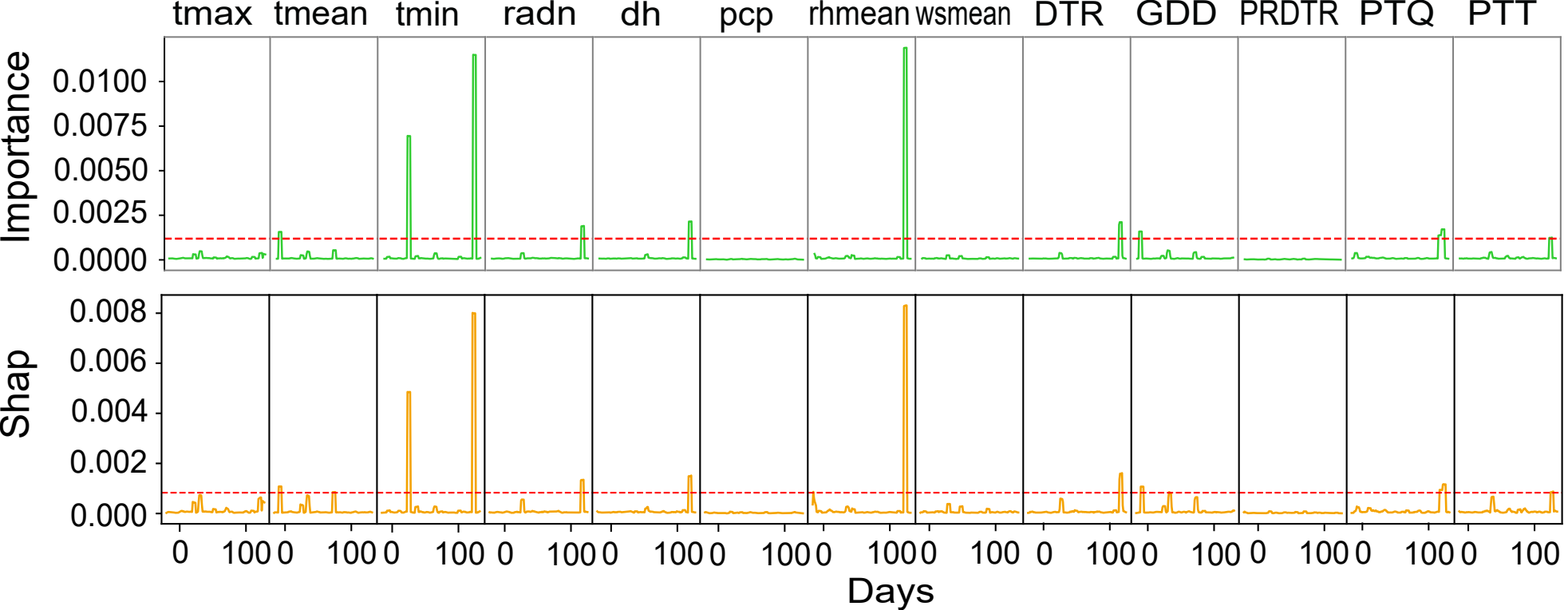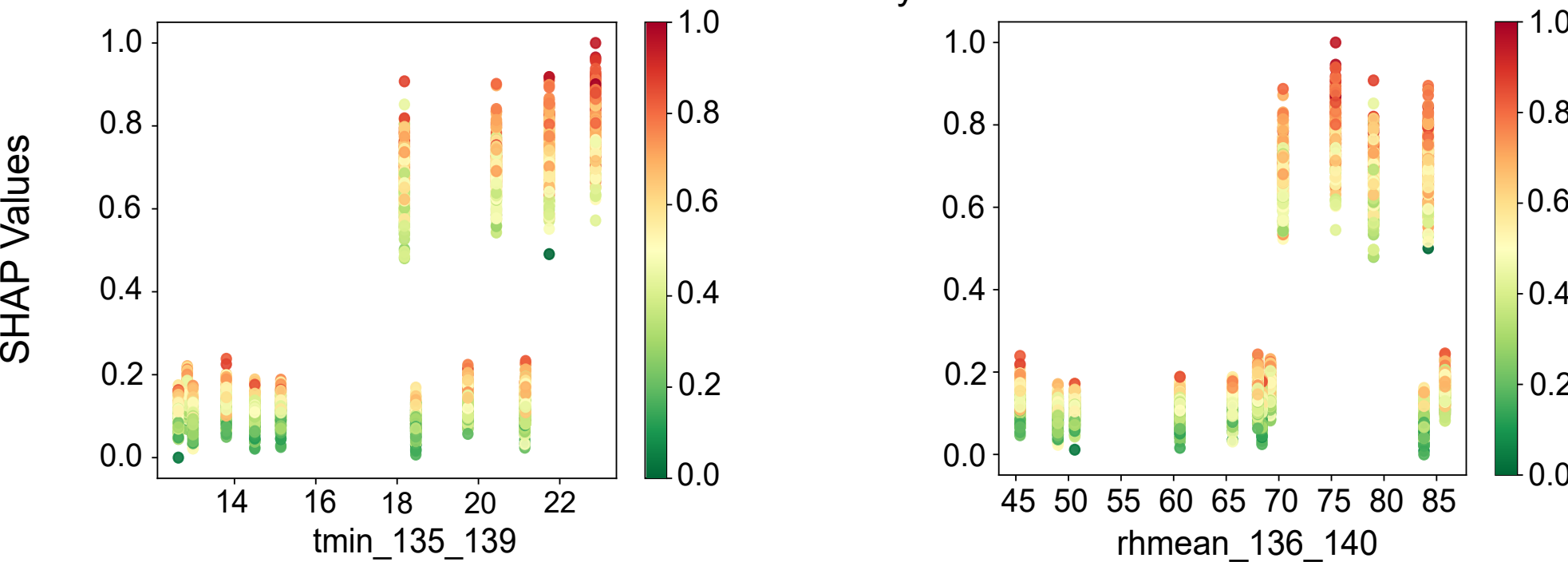

Supplement: Supplementary file 1 [file plants-14-02053-s001.zip › fig_S10.pdf]

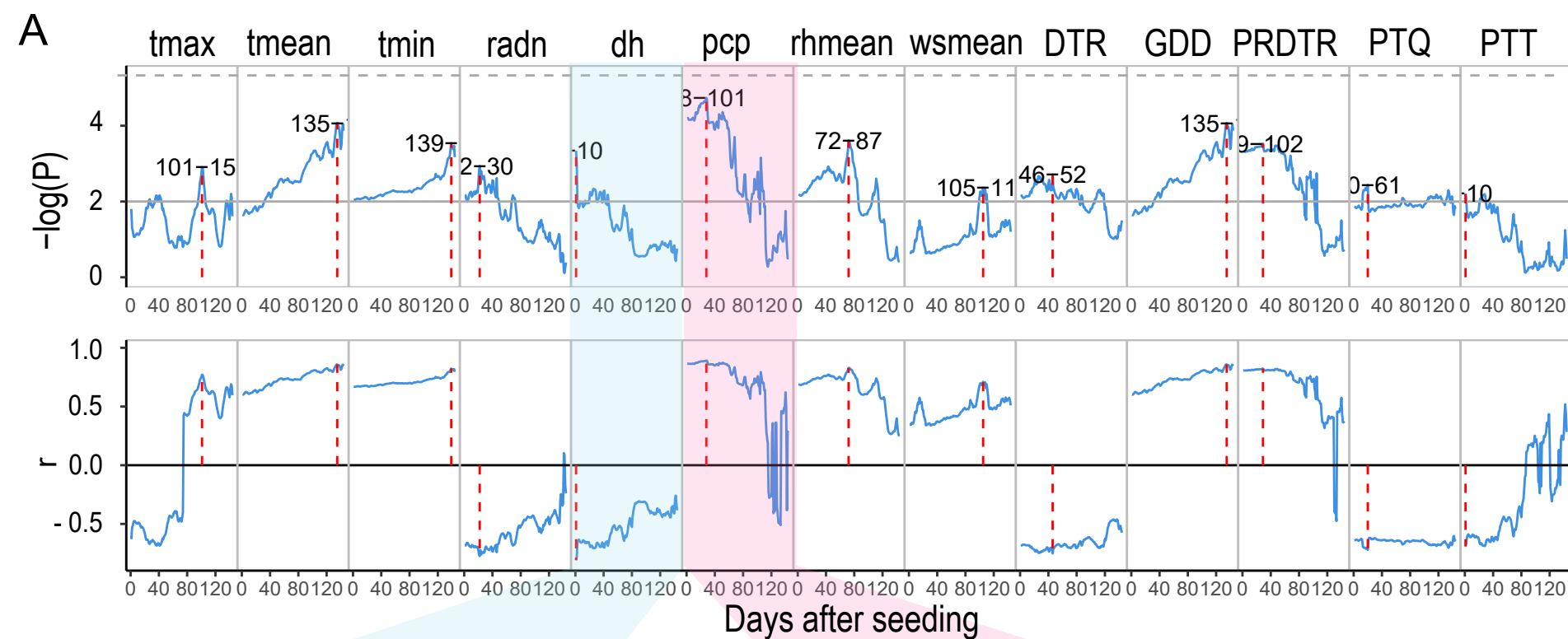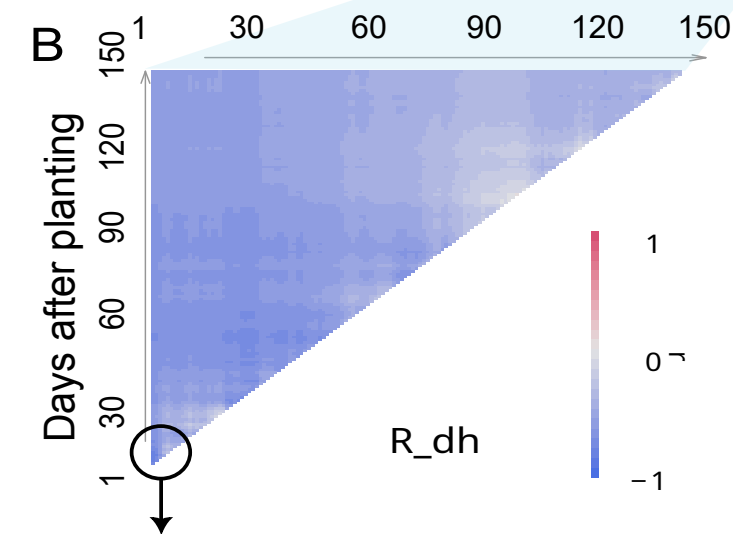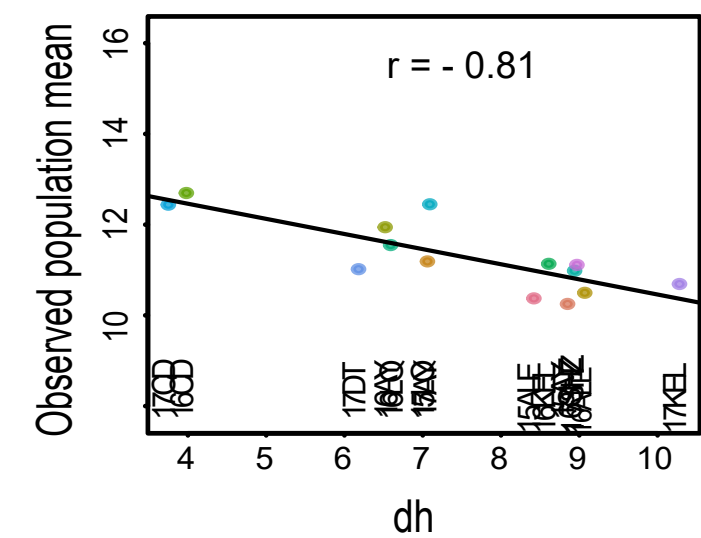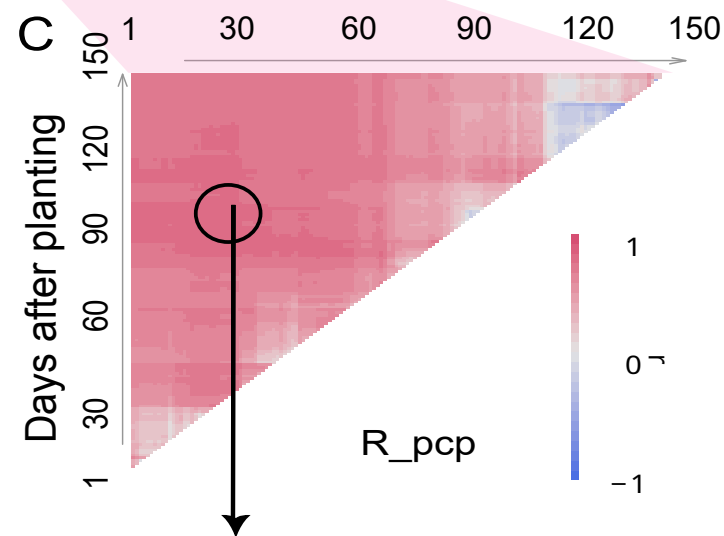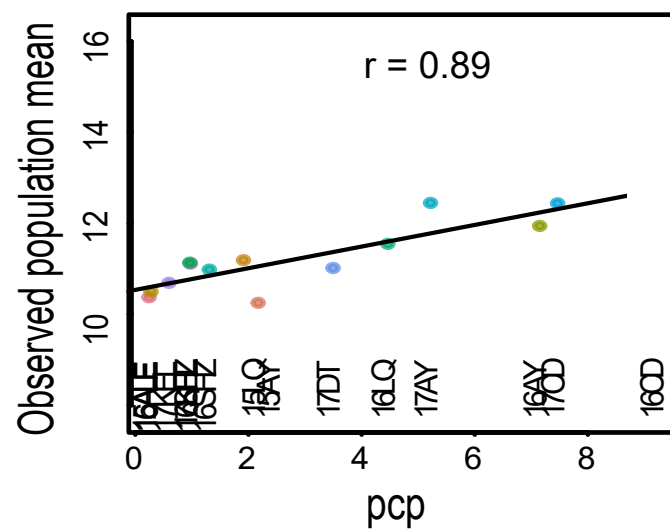

Supplement: Supplementary file 1 [file plants-14-02053-s001.zip › fig_S3.pdf]

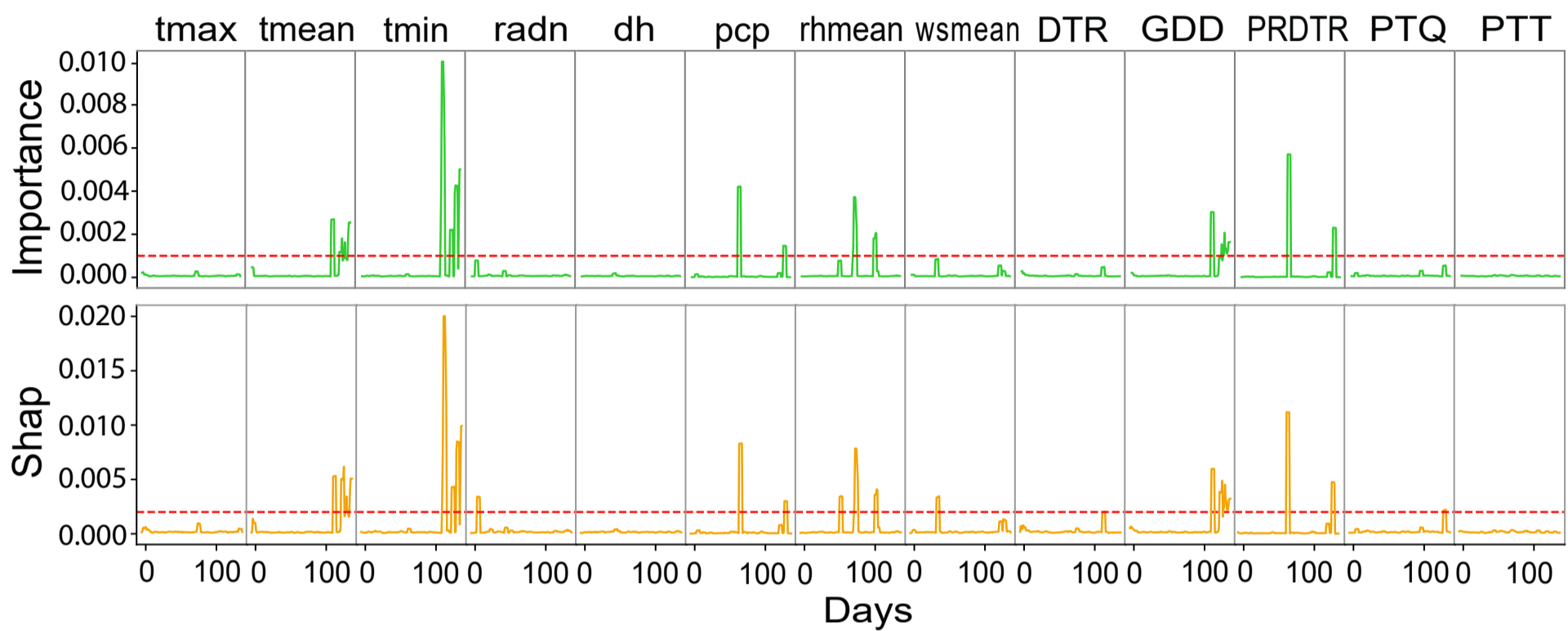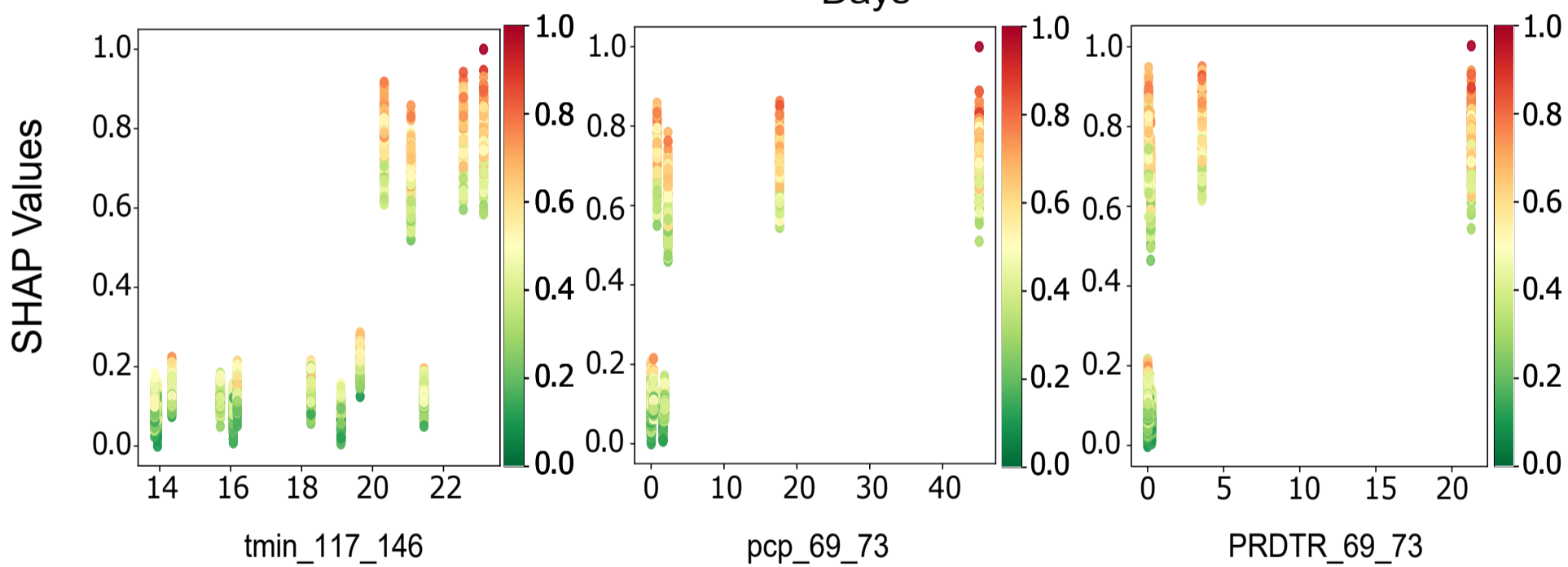

Supplement: Supplementary file 1 [file plants-14-02053-s001.zip › fig_S4.pdf]

**A**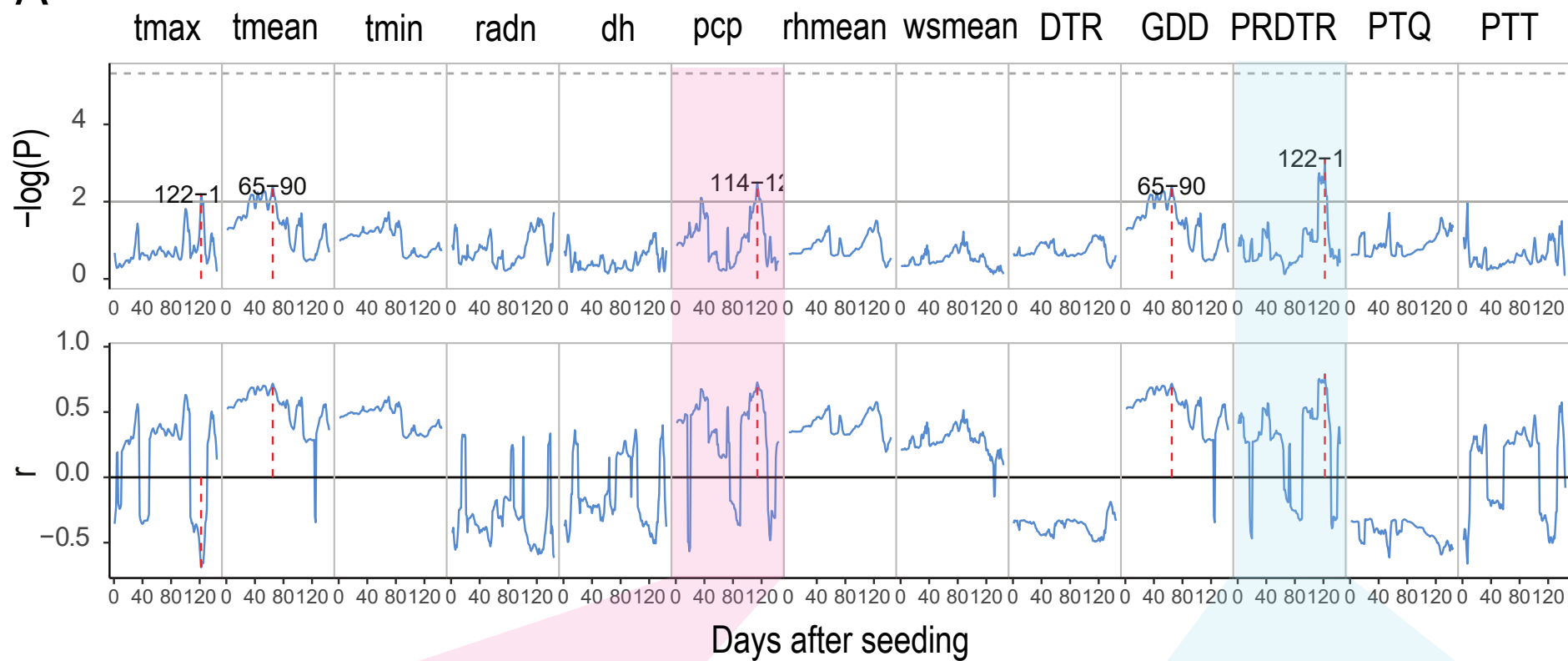**B**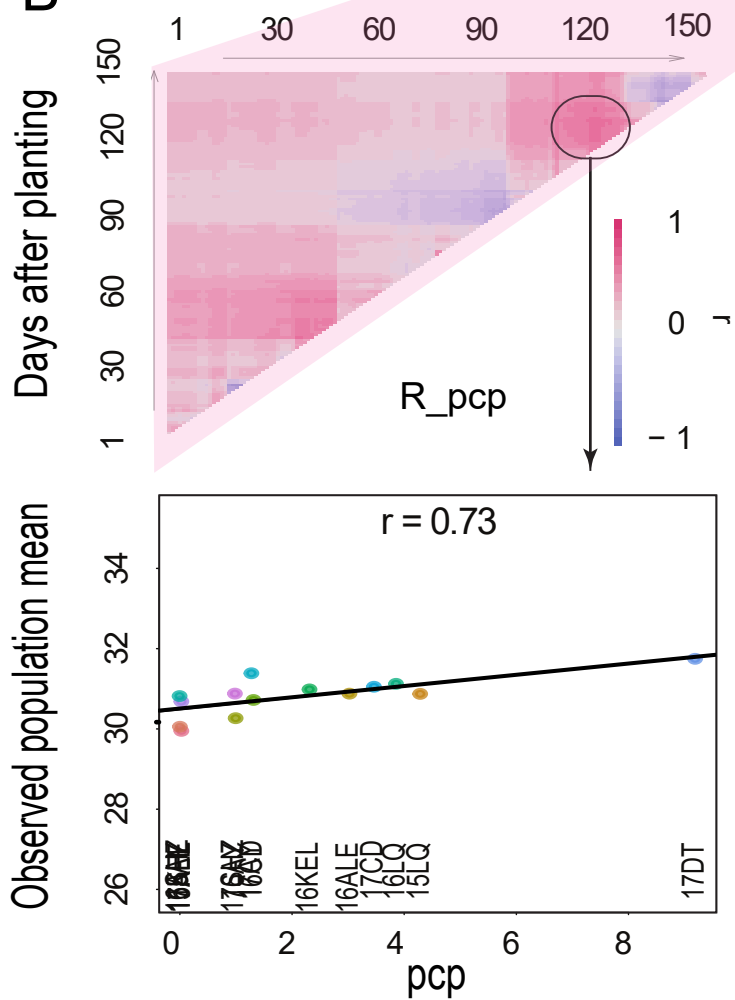**C**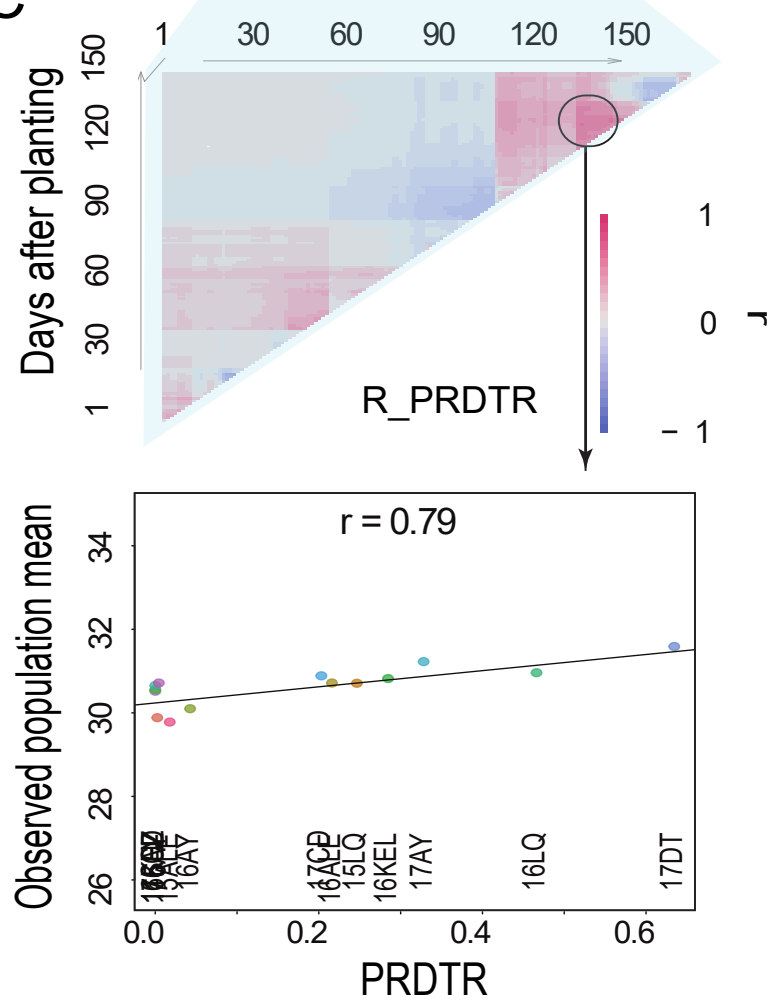

Supplement: Supplementary file 1 [file plants-14-02053-s001.zip › fig_S5.pdf]

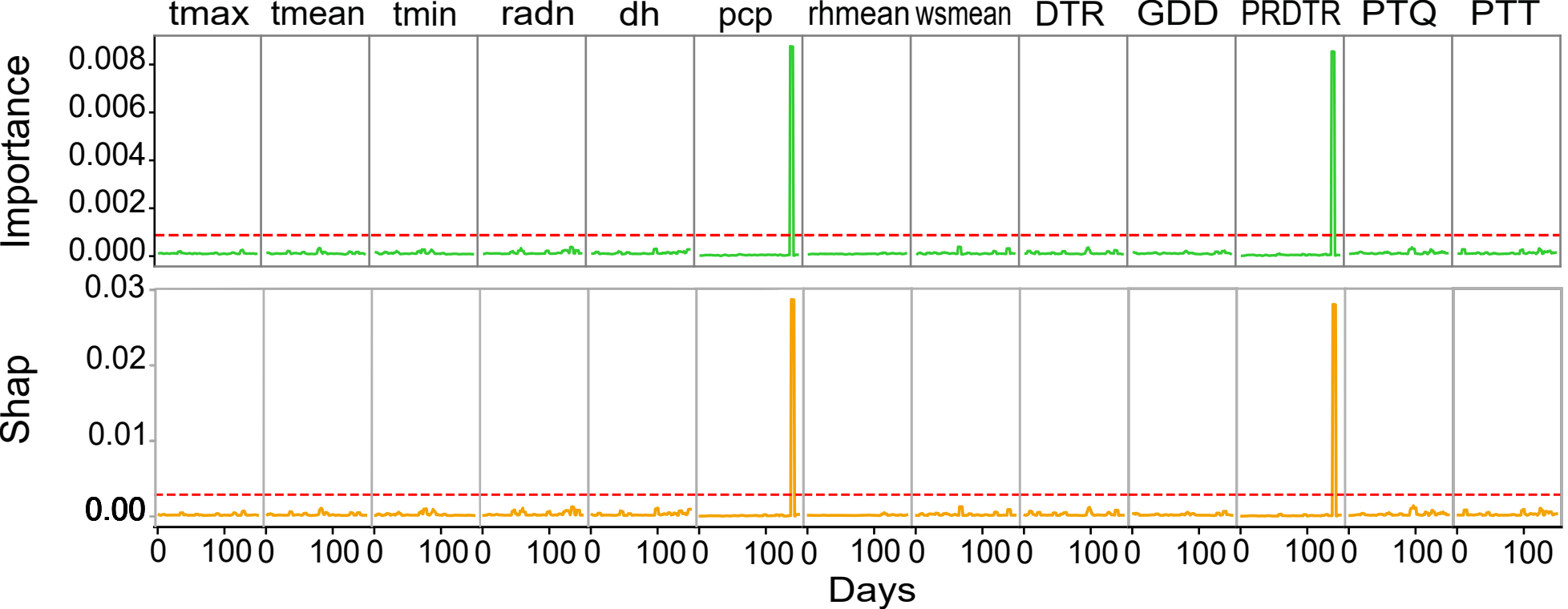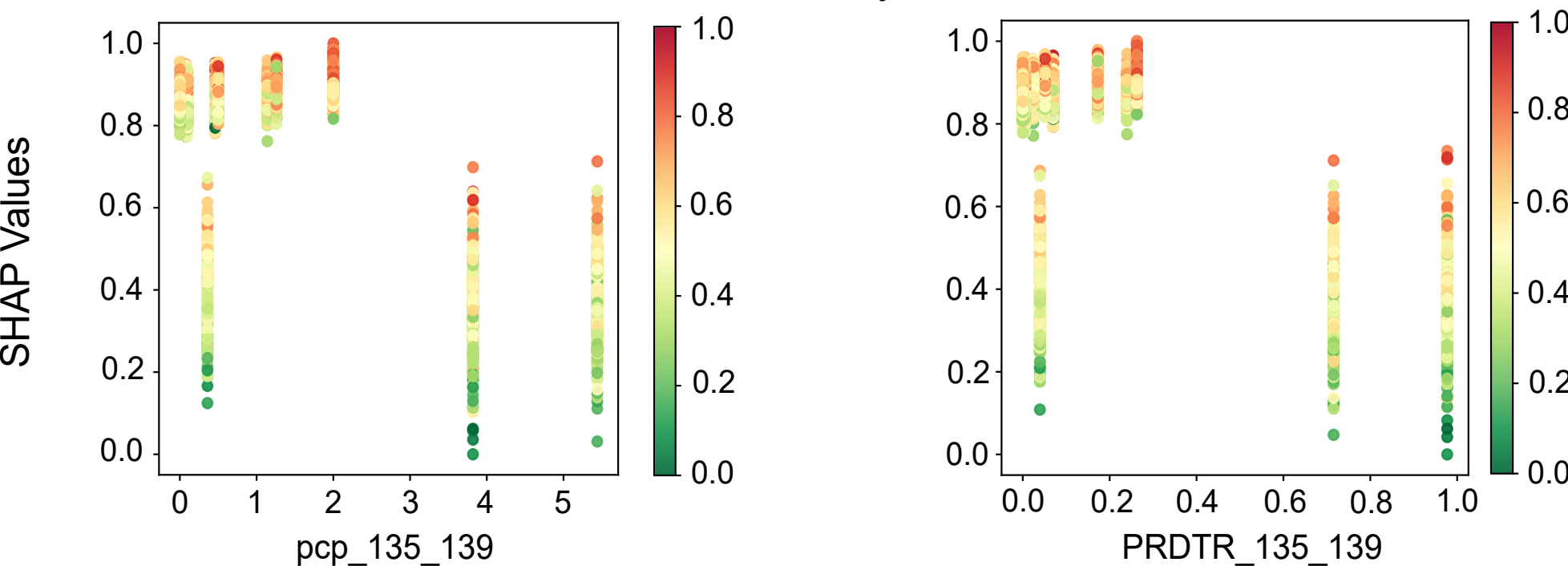

Supplement: Supplementary file 1 [file plants-14-02053-s001.zip › fig_S6.pdf]

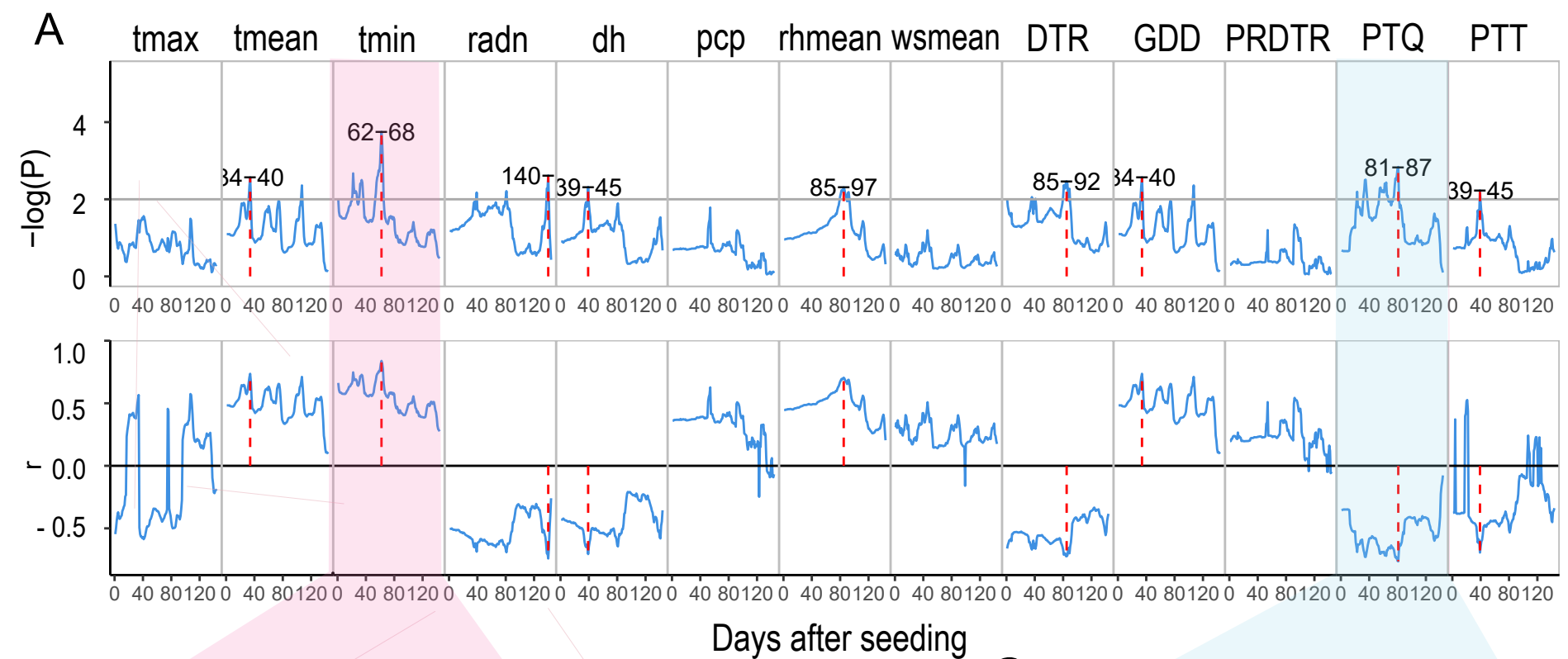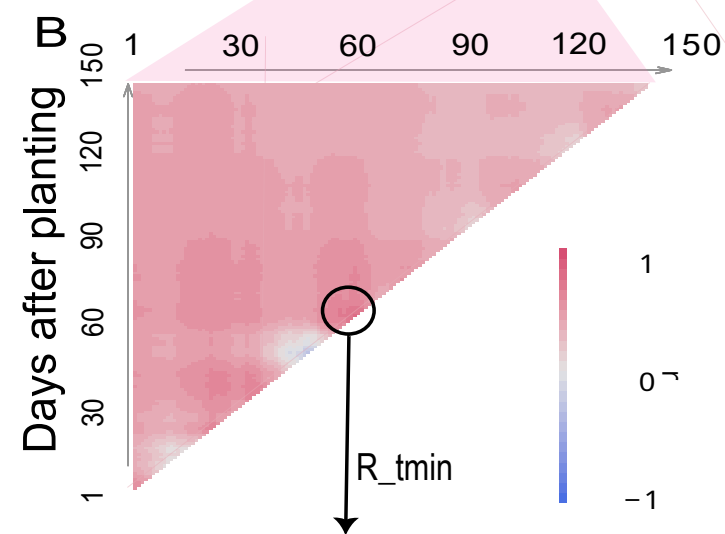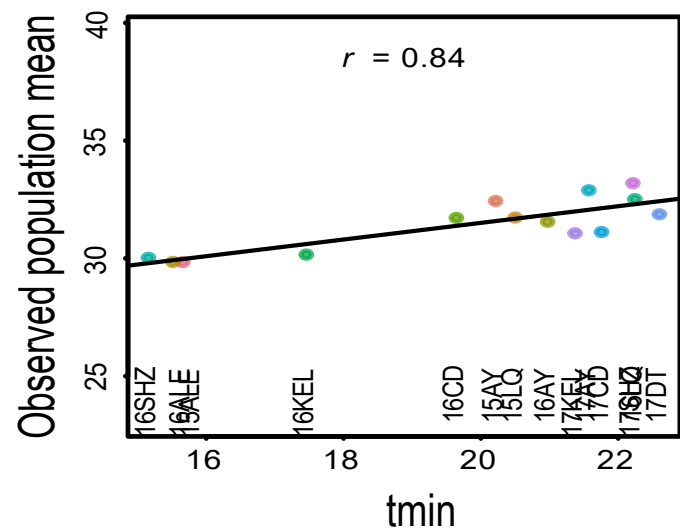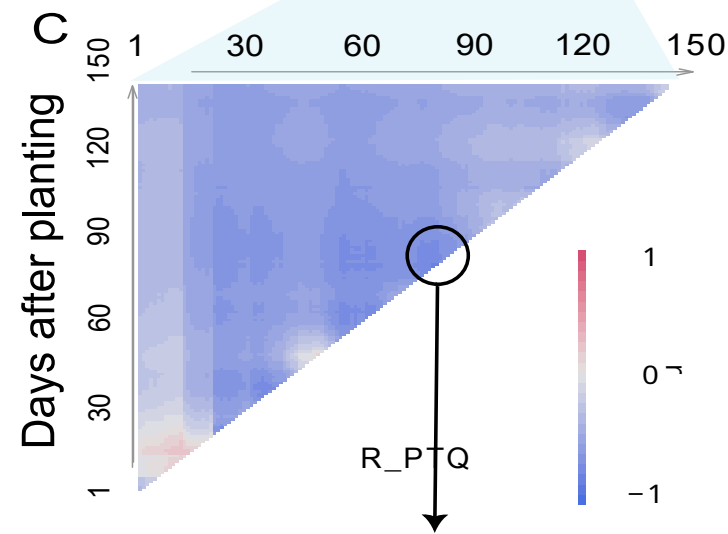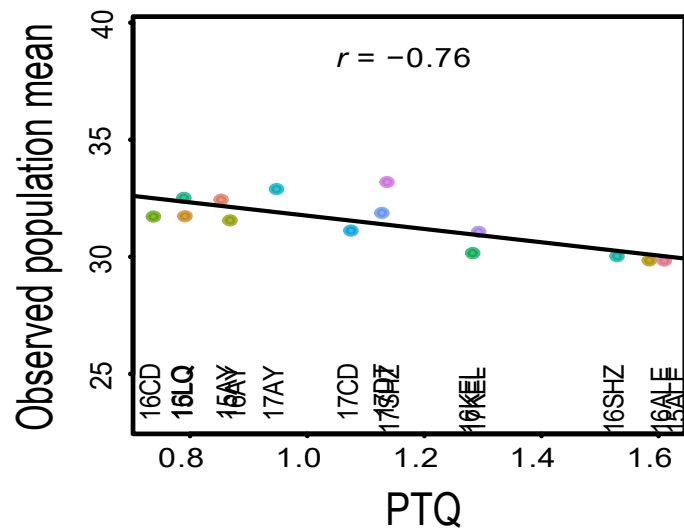

Supplement: Supplementary file 1 [file plants-14-02053-s001.zip › fig_S7.pdf]

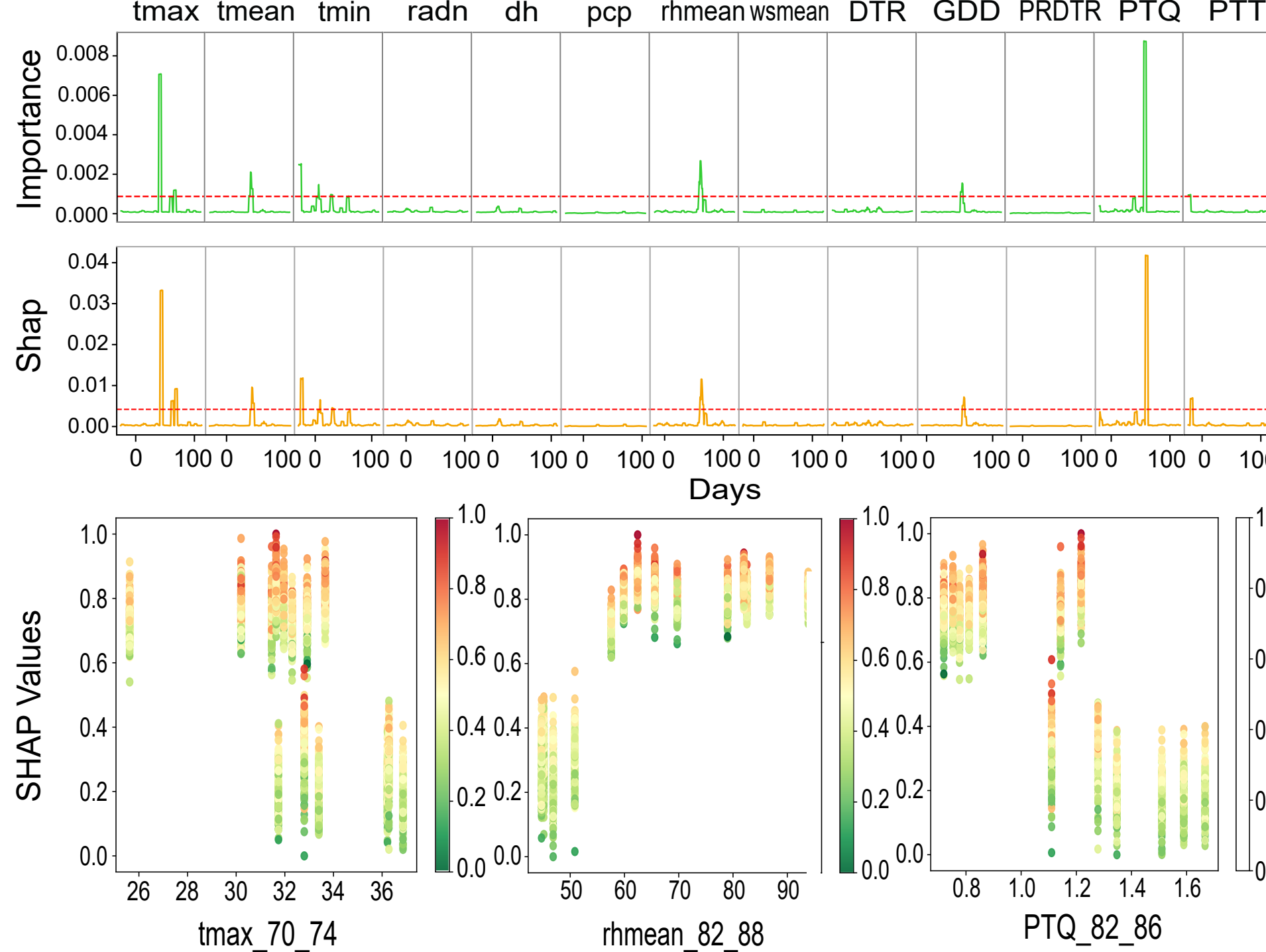

Supplement: Supplementary file 1 [file plants-14-02053-s001.zip › fig_S8.pdf]
