# Supplementary material for: Machine Learning-Driven Identification of Key Environmental Factors Influencing Fiber Yield and Quality Traits in Upland Cotton
Source: Plants (Basel). 2025 Jul 4;14(13):2053. doi: 10.3390/plants14132053 (PMC12252131; doi:10.3390/plants14132053)
Supplement: Supplementary file 1 [file fig_S2.pdf › fig_S2.pdf]

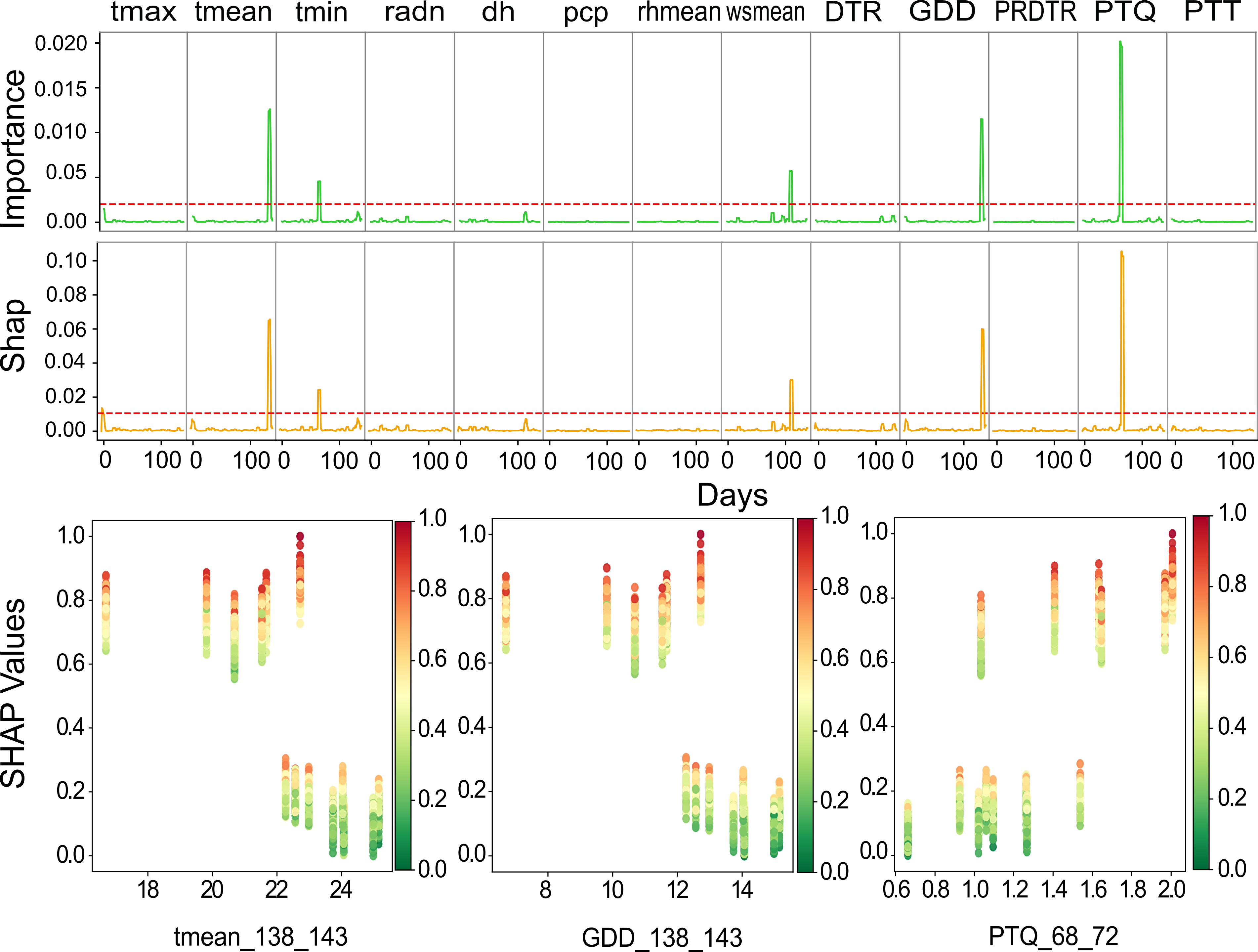

tmax
tmean
tmin
radn
dh
pcp
rhmean
wsmean
DTR
GDD
PRDTR
PTQ
PTT
Importance
0.020
0.015
0.010
0.05
0.00
Shap
0.10
0.08
0.06
0.04
0.02
0.00
0
100
Days
SHAP Values
1.0
0.8
0.6
0.4
0.2
0.0
18
20
22
24
tmean_138_143
8
10
12
14
GDD_138_143
0.6
0.8
1.0
1.2
1.4
1.6
1.8
2.0
PTQ_68_72
