# Supplementary material for: Machine Learning-Driven Identification of Key Environmental Factors Influencing Fiber Yield and Quality Traits in Upland Cotton
Source: Plants (Basel). 2025 Jul 4;14(13):2053. doi: 10.3390/plants14132053 (PMC12252131; doi:10.3390/plants14132053)
Supplement: Supplementary file 1 [file fig_S9.pdf › fig_S9.pdf]

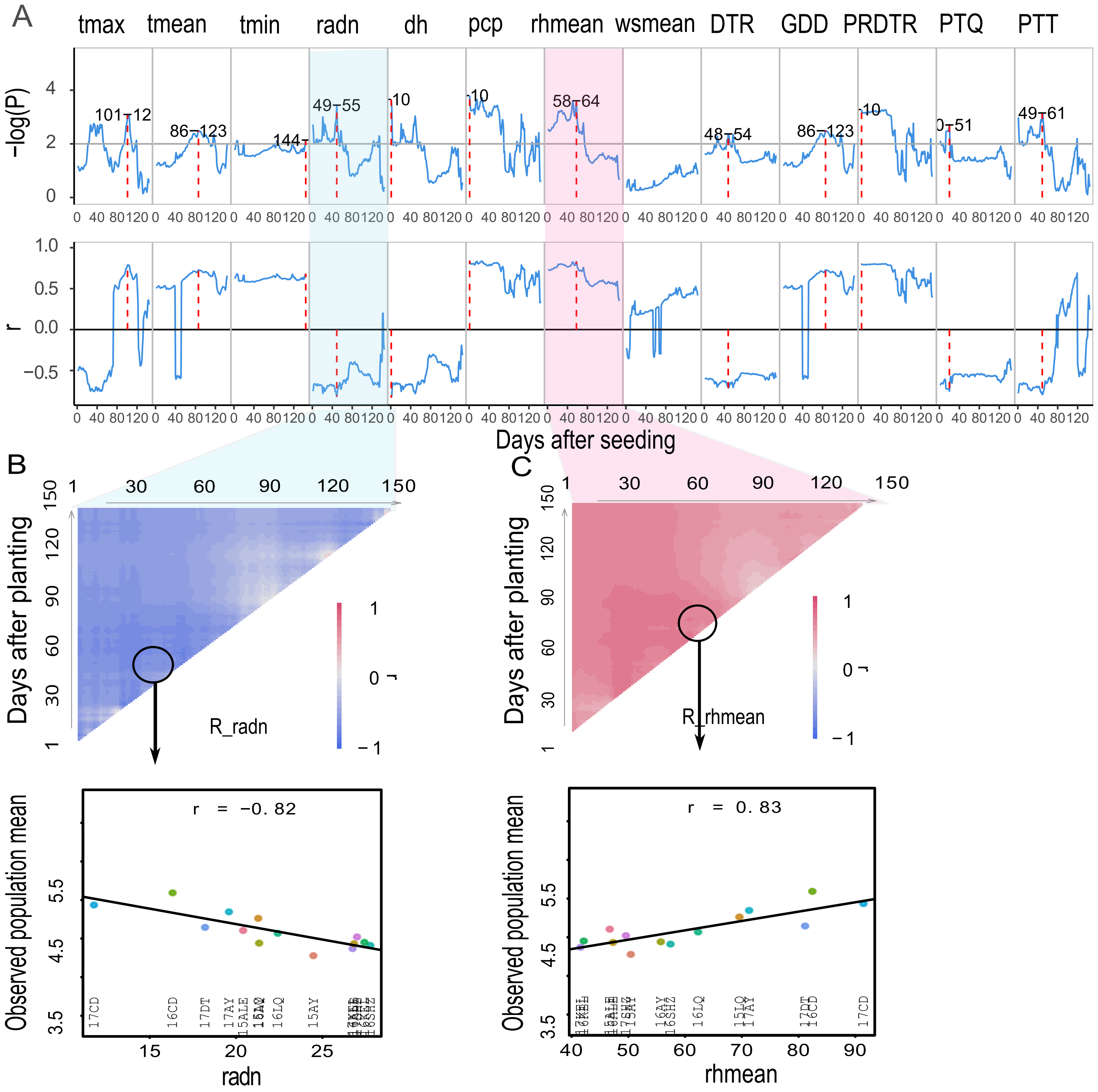
A
tmax
tmean
tmin
radn
dh
pcp
rhmean
wsmean
DTR
GDD
PRDTR
PTQ
PTT
-log(P)
101-12
86-123
144
49-55
10
10
58-64
48-54
86-123
10
0-51
49-61
r
Days after seeding
B
C
Days after planting
R_radn
R_rhmean
r = -0.82
r = 0.83
Observed population mean
radn
rhmean
